# A chromosome-level genome assembly of the soybean pod borer: insights into larval transcriptional response to transgenic soybean expressing the pesticidal Cry1Ac protein

**DOI:** 10.1186/s12864-024-10216-2

**Published:** 2024-04-09

**Authors:** Yangzhou Wang, Yao Yao, Yunyue Zhang, Xueyan Qian, Dongquan Guo, Brad S. Coates

**Affiliations:** 1 Jilin Academy of Agricultural Sciences, Changchun, 130033 China; 2 United States Department of Agriculture, Agricultural Research Service, Corn Insects & Crop Genetics Research Unit, 532 Science II, 2310 Pammel Dr., Ames, IA 50011 USA

**Keywords:** *Leguminivora glycinivorella*, Genome, Transcriptome, *Glycine max*, *Bacillus thuringiensis*, Cry1Ac pesticidal protein

## Abstract

**Background:**

Genetically modified (GM) crop plants with transgenic expression of *Bacillus thuringiensis* (Bt) pesticidal proteins are used to manage feeding damage by pest insects. The durability of this technology is threatened by the selection for resistance in pest populations. The molecular mechanism(s) involved in insect physiological response or evolution of resistance to Bt is not fully understood.

**Results:**

To investigate the response of a susceptible target insect to Bt, the soybean pod borer, *Leguminivora glycinivorella* (Lepidoptera: Tortricidae), was exposed to soybean, *Glycine max*, expressing Cry1Ac pesticidal protein or the non-transgenic parental cultivar. Assessment of larval changes in gene expression was facilitated by a third-generation sequenced and scaffolded chromosome-level assembly of the *L. glycinivorella* genome (657.4 Mb; 27 autosomes + Z chromosome), and subsequent structural annotation of 18,197 RefSeq gene models encoding 23,735 putative mRNA transcripts. Exposure of *L. glycinivorella* larvae to transgenic Cry1Ac *G. max* resulted in prediction of significant differential gene expression for 204 gene models (64 up- and 140 down-regulated) and differential splicing among isoforms for 10 genes compared to unexposed cohorts. Differentially expressed genes (DEGs) included putative peritrophic membrane constituents, orthologs of Bt receptor-encoding genes previously linked or associated with Bt resistance, and those involved in stress responses. Putative functional Gene Ontology (GO) annotations assigned to DEGs were significantly enriched for 36 categories at GO level 2, respectively. Most significantly enriched cellular component (CC), biological process (BP), and molecular function (MF) categories corresponded to vacuolar and microbody, transport and metabolic processes, and binding and reductase activities. The DEGs in enriched GO categories were biased for those that were down-regulated (≥ 0.783), with only MF categories GTPase and iron binding activities were bias for up-regulation genes.

**Conclusions:**

This study provides insights into pathways and processes involved larval response to Bt intoxication, which may inform future unbiased investigations into mechanisms of resistance that show no evidence of alteration in midgut receptors.

**Supplementary Information:**

The online version contains supplementary material available at 10.1186/s12864-024-10216-2.

## Background

The development of resistance to insecticidal agents by insect pests is a threat to sustainable agricultural production practices [1, 2] and human health [3]. The number of insect species with resistance to one or more insecticides has increased rapidly since the post-World War II development and subsequent widespread use of broadcast synthetic chemical agents [4, 5]. These resistant phenotypes result from mutations that disrupt the insecticidal mode of action (MoA), including those that alter the kinetics of target receptor binding, increase rates of detoxification and excretion, or cause behavioral avoidance [6]. Genetically modified (GM) cotton and maize that express one or more pore-forming 3-domain crystalline (Cry) pesticidal proteins or vegetative insecticidal protein 3A (Vip3A) derived from *Bacillus thuringiensis* (Bt) [7] are currently planted on most cultivated hectares in North and South America [8, 9]. Field populations of target insect pests have henceforth evolved practical resistance to crops that express Bt proteins [10]. Bt resistance in field populations has occurred despite implementation of insect resistance management (IRM) plans which rely on high-dose refuge (HDR) strategies [11–13]. Resistance arguably occurs when one or more assumptions of the HDR strategy are violated [14], and include instances when resistance alleles are non-recessive [15, 16], insects feed on plant tissues with a dose of Bt that does not cause mortality among heterozygous genotypes carrying one resistance allele resulting in functional resistance [17–19], or resistant phenotypes lacked fitness costs that were proposed to delay the onset of widespread resistance [20, 21]. These factors raise concerns for the long-term sustainability and efficacy of Bt technology [22].

The proposed MoA for Cry1A proteins includes the sequential binding model where pesticidal proteins bind membrane-anchored midgut receptors that potentiate the formation of pore channels that in-turn cause cell swelling and death via osmotic imbalance [23, 24]. Additionally, a signal transduction model proposes intracellular signaling and induction of the protein kinase A (PKA) pathway causes cell death (apoptosis) [25, 26]. Among species of Lepidoptera, resistance in selected laboratory colonies and field populations is associated with structural or functional changes in midgut receptors [27], including ATP binding cassette (ABC) transporters [28, 29], cadherin, tetraspanin, alkaline phosphatase and aminopeptidase N [30]. Genes in the mitogen-activated protein kinase (MAPK) pathway also have been linked or associated with resistance [31–34], wherein this pathway may impact the expression of Bt receptors themselves [35]. Additional evidence suggests that up-regulation of genes encoding constituents of insect peritrophic membranes may decrease susceptibility when exposed to Bt proteins [36, 37] or Bt bacterial infections [38]. Additionally, increased proteolytic degradation of Bt in the gut or repair mechanisms have been proposed [30].

The efficacy of the HDR strategy and other IRM tactics may arguably be enhanced through the greater understanding of the molecular mechanisms involved in resistance [1]. Furthermore, cellular responses to Bt intoxication even in susceptible insects may provide insights into Bt MoA and points at which disruption might lead to resistance [39]. Among such comparisons between susceptible or resistant insects exposed or unexposed to Bt proteins, hundreds or thousands of transcripts are often differentially expressed [39–47]. In some instances, differentially expressed transcripts are enriched for those putatively functioning in general stress response pathways (e.g. cytochrome P450 monooxygenases, esterases, oxidases, and peroxidases) and having transporter function [45], involved in cell survival mechanisms [39] or cell repair and immune function [47]. Additionally, orthologs and paralogs of genes putatively functioning as Bt receptors in the insect midgut (cadherin, aminopeptidases N and ABC transporters) are differentially expressed between Bt intoxicated susceptible insects compared to unintoxicated cohorts [39, 43, 45]. Role of these genes in response to intoxication remains uncertain, but may be informed following further studies [39].

The soybean pod borer, *Leguminivora glycinivorella* (Matsumura) (Lepidoptera: Tortricidae), is a destructive agricultural pest insect of cultivated soybean, *Glycine max* (Fabales: Fabaceae) (L.) [48]. This insect has a univoltine life cycle with mature 4th instars that enter the soil and form cocoons in hollowed chambers during mid- to late-September wherein they diapause. After approximately 8–9 months larvae pupate and emerge as adults in early summer [49]. Female oviposition and larval *L. glycinivorella* feeding primarily occurs on a narrow range of leguminous plants including *G. max*, wild soybean, *G soja*, and the shrubby relative of pea, *Sophora flavescens*. Larval feeding primarily occurs on the pods of *G. max*, which causes substantial reductions in grain yield and quality [50, 51]. High infestations can lead to 10–30% yield loss and exceed 50% during severe outbreaks [52]. The endemic geographic range of this species includes Japan and Korea, and across eastern China. The most severe outbreaks tend to be in the northeastern Heilongjiang, Jilin, and Liaoning Provinces, which coincides with areas where approximately two-thirds of soybeans are grown in China [53], making *L. glycinivorella* a primary pest of this crop. Although foliar insecticides are applied, effective contact and reduction in feeding damage is difficult to achieve due to protection of larva under *G. max* leaf canopies and within pods [54]. Use of systemic insecticides can lead to more consistent reductions in *L. glycinivorella* feeding damage, but ecological concerns limit their use especially given evidence of impacts on pollinator health [55].

Cultivars of *G. max* selected for host plant traits can reduce levels of *L. glycinivorella* feeding damage [56], but do not always prevent significant economic loss to growers. Transgenic expression of insecticidal molecules by *G. max* show promise as an *L. glycinivorella* control tactic. For instance, damage is reduced on plants that express double stranded RNA (dsRNA) that elicits an RNA interference (RNAi)-based knockdown of a serine protease in *L. glycinivorella* larvae [57, 58]. Pesticidal Bt proteins expressed by transgenic *G. max* reduce damage by several lepidopteran pests [59–61]. Commercial use of Bt cotton expressing the Bt Cry1Ac protein was approved by China in 1997 [62]. Since 2016 Chinese government directives aim to commercialize GE cotton, maize and soybean for domestic use [63] and has culminated in recent changes that allow for their widespread use [64]. Bt IRM strategies for cotton pests have been established in China [65]. Investigations into non-target effects and ecological assessments of Bt crops [66] and target insect baseline Bt susceptibilities have also been conducted pre-commercialization for many crops in China [67]. However, investigation into potential for Bt resistances among many target pests is lacking.

In this study, we used *L. glycinivorella* as a model to determine the effects of Bt intoxication on larval gene expression following exposures to a transgenic *G. max* cultivar that expresses the Bt Cry1Ac pesticidal protein [61], informing future research into Bt MoA and potential mechanisms of resistance. This was accomplished by predicting differentially expressed genes (DEGs) between susceptible *L. glycinivorella* larvae feeding for 2 days on Bt Cry1Ac compared to conventional non-Bt cultivars. An annotated chromosome-level genome assembly for *L. glycinivorella* was generated and provided resources to investigate transcriptional effects of sublethal Bt exposures. Changes in gene expression among target insects following exposure to Bt transgenic crops, such as those documented in this study, provide insights into cellular responses. Such insight may also inform future research regarding potential adaptive mechanisms that lead to field-evolved resistance.

## Results

### Genomic library construction, sequencing, and read filtering

**Fig. 1:**
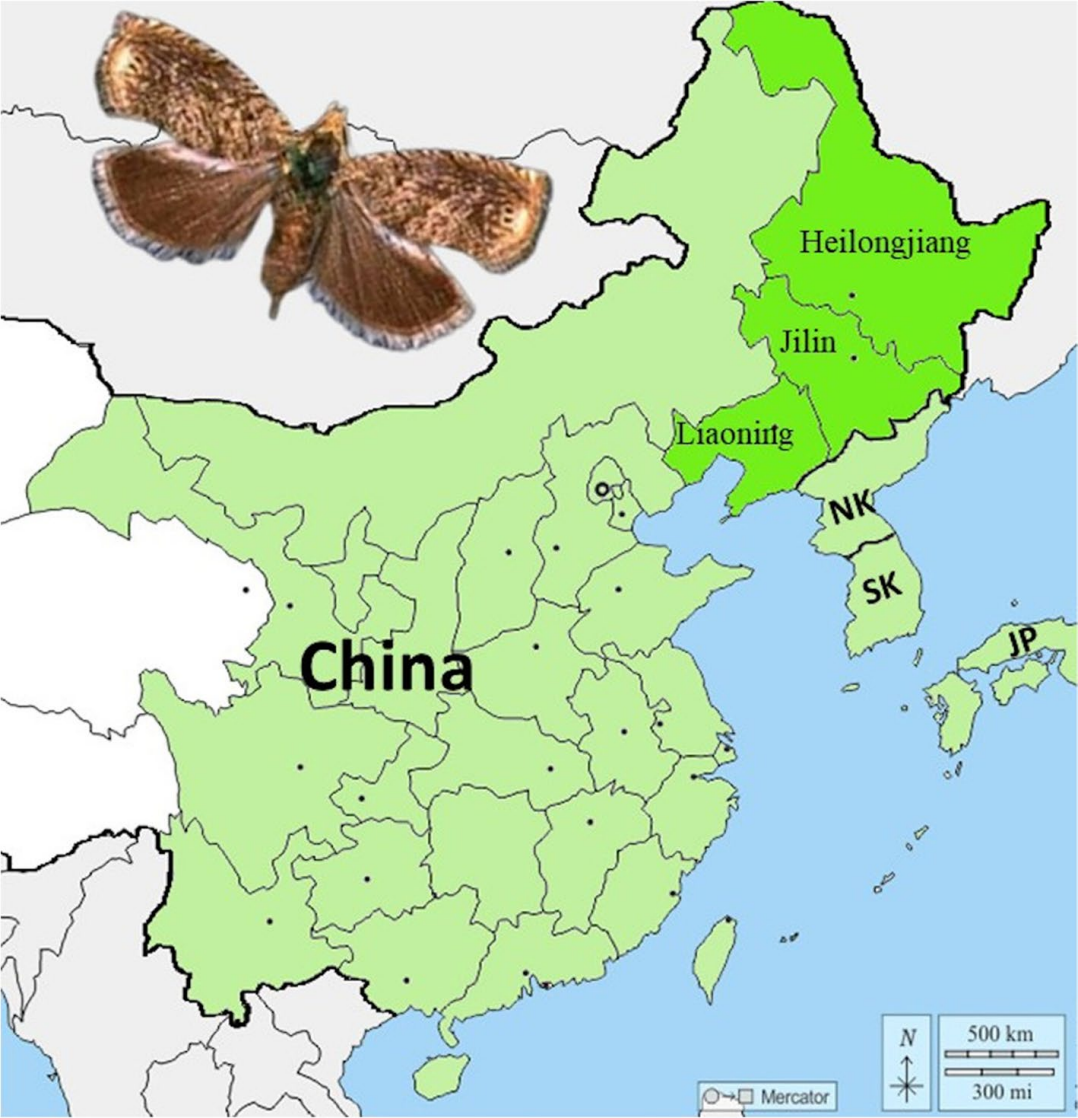
Approximate geographic distribution of *Leguminivora glycinivorella* across China, North Korea (NK), South Korea (SK), and Japan (JP; partial). Areas with greatest damage to cultivated soybean, *Glycine max*, indicated in darker green. Location of collected BioSample SAMN21160035 used in genome assembly is indicated by red pin

Output from the Oxford Nanopore PromethION P48 provided 6.4 million reads spanning 66.6 Gb of raw read data for library LglyNP generated from a single male larva collected in Jilin Province, China (Fig. 1) (Table S1; N50 = 19.6 kb L50 = 1.1 M), of which 61.6 Gb among 5.7 million reads remained post-filtering (N50 = 10.8 kb L50 = 1.0 M). A high proportion of short reads failed to surpass the *q* > 7 cutoff (Fig S1A), but the number of filtered bases was less skewed (Fig S1B). The final set of filtered reads had a mean *q* = 12.35 (Fig S1C) with read lengths up to 246.3 kb. A total of 386.7 M short 150 bp PE reads were generated covering 58.0 Gb of nucleotide data (Table S1) of which 97.29% had a *q* ≥ 20. All raw genomic read data are available in the NCBI Sequence Read Archive (SRA; Table S1) under BioProject PRJNA759210.

### K-mer based genome size estimates

**Fig. 2:**
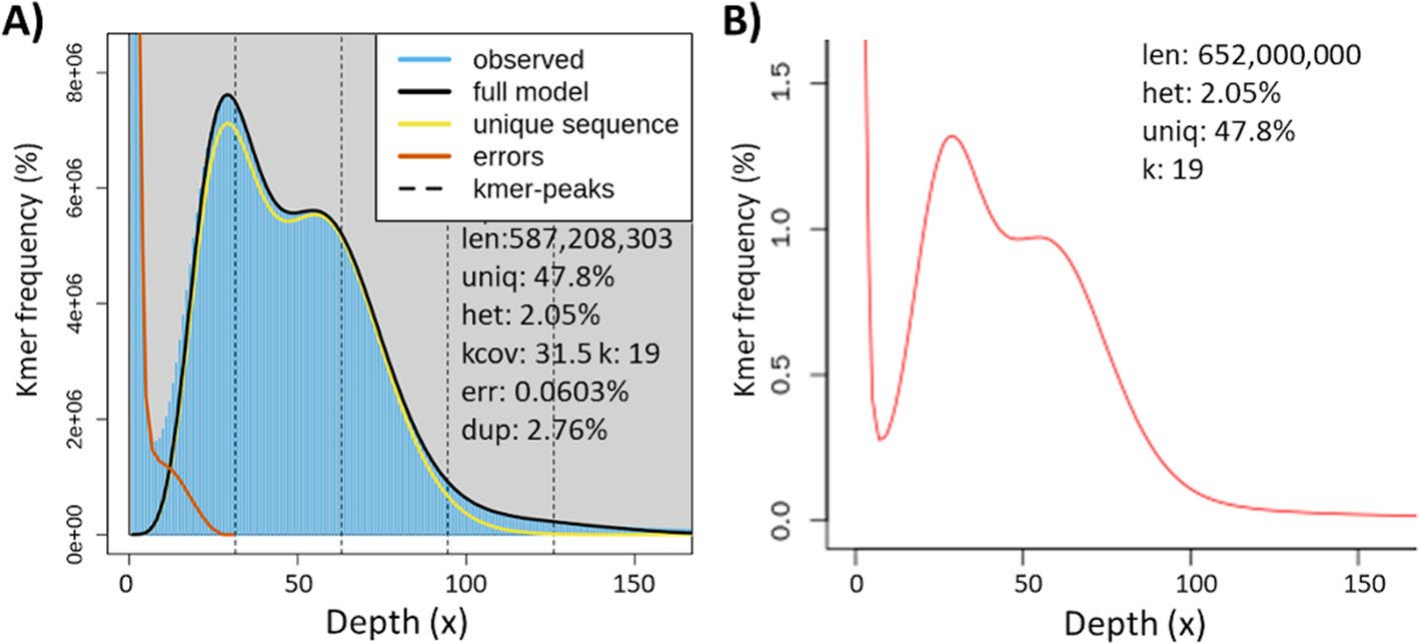
Kmer-based estimates of *Leguminivora glycinivorella* genome size (len), percent unique non-repetitive portion (uniq), heterozygous (het) and duplicated (dup) fractions, and erroneous sequence (err) from **A** GenomeScope 2.0 and **B** Genome Character Estimator (GCE) analyses

Both GCE and GenomeScope2 output showed a frequency distribution with a large number with low coverages at kmer = 19 representing sequencing error within the short Illumina PE reads, along with a homozygous (1 N) peak at a kmer depth of 60 (Fig. 2). The greater frequency of different kmers in the peak at a depth of 36 represented the heterozygous portion for this diploid organism. The two additional peaks at 92 and 126 corresponding to duplicated heterozygous and homozygous fractions, respectively. Genome size estimates differed between GenomeScope2 (587.2 Mb; Fig. 2A) and GCE (652 Mb; Fig. 2B) while percentage unique sequence remained consistent at 47.8% (52.8% repetitive). An estimated heterozygosity of 2.05% was obtained from both GenomeScope2 and GCE.

### Contig assembly

A final set of 225 contigs were obtained from the initial NextDenovo assembly of Illumina and Oxford Nanopore reads after duplicates were purged and error corrected using PE short read data. Contigs in this 657.4 Mb *L. glycinivorella* haploid assembly, ilLegGlyc1.0, ranged from 0.13 to 37.63 Mb in size with an N50 of 4.2 Mb (Table 1) and contained no gaps (Ns). Alignment of filtered short PE 150 bp reads to these 225 contigs resulted in 99.74% coverage at a mean depth of 85.48-fold among the 99.3% of reads that aligned. BUSCO assessment of genome completeness as gauged by representation of the lepidoptera_odb10 set of orthologs (Table 2) indicated 92.9% were complete [91.0% single copy (S) and 1.9% duplicated (D)] and 0.8% fragmented BUSCOs (F), while 6.3% were missing (M).

**Table 1:** Metrics for the *Leguminivora glycinivorella* genome assembly, ilLegGlyc1.1

Metric	ilLegGlyc1.x
Contig assembly: ilLegGlyc1.0	
Total number	240
Length (bp)	657,403,812
N50 (Mb)	4,159,467
L50	49
N90 (bp)	1,616,361
L90	148
Largest contig (bp)	37,635,290
Scaffold assembly: ilLegGlyc1.1^a^	
Total number	40
Length (bp)	657,423,512
GC content (%)	
Gaps	197
N bases (within gaps)	19,700
N50 (bp)	25,267,037
L50	11
N90 (bp)	15,140,355
L90	23
Largest scaffold (bp)	55.408,830
Gene Annotations^b^	
RefSeq gene models (total)	18,197
Protein-coding	15,681
Non-coding	2,382
Pseudogenes	134
lncRNAs	1,042
rRNAs	75
tRNAs	912
snRNAs, snoRNAs, misc_RNAs	473
RefSeq mRNA models (XM_)	23,735
Fully supported^c^	21,155
RefSeq protein models (XP_)	23,735
Repeat content (masked sequence)	48.7%

^a^ *Leguminivora glycinivorella* WGS Project: JAKXMO01; GenBank assembly accession: GCA_023078275.1* (this assembly)*

^b^RefSeq assembly accession: GCF_023078275.1

^c^100% of length with transcript evidence; 800 million reads (https://www.ncbi.nlm.nih.gov/genome/annotation_euk/Leguminivora_glycinivorella/100/#AlignmentStats)

**Table 2:** Comparison of the *Leguminivora glycinivorella* genome assembly to those from Tortricid moths (Lepidoptera: Tortricidae). Completeness assessed using the Benchmarking Universal Single-Copy Orthologs (BUSCO) against the lepidoptera_odb10. Set of 5,286 orthologs

	Assemblies of tortricid moth species					
**Statistics**	ilLegGlyc1.0	ilLegGlyc1.1	ilCydSple1.2	PamFasc1.1	NotUddm1.1	ApoTurb1.1
Contig or scaffold no	225	40	112	34	49	44
Chromosomes	Na	27 + Z	27 + Z	27 + Z	27 + Z	27 + Z
Length (Mb)	657.4	657.4	630.62	564.4	794.12	720.47
N50 (Mb)	4.2	25.3	22.1	20.7	30.0	27.1
L50	49	11	12	11	11	11
N90 (Mb)	148	15.1	26	24	23	24
L90	1.6	23	11.6	13.0	17.7	16.5
Longest (Mb)	37.6	55.4	49.8	46.7	75.7	61.5
**BUSCOs**	LegGlyc1.0	LegGlyc1.1	CydSple1.1	PamFasc1.1	NotUddm1.1	ApoTurb1.1
Complete ©	4912; 92.2%	4914; 93.0%	5167; 97.8%	5189; 98.2%	5197; 98.3%	5194; 98.2%
Complete single-copy (S)	4812; 91.0%	4837; 91.5%	5136; 97.2%	5143; 97.3%	5159; 97.6%	5156; 97.5%
Complete duplicated (D)	100; 1.9%	77; 1.5%	31; 0.6%	46; 0.9%	38; 0.7%	38; 0.7%
Fragmented BUSCOs (F)	44; 0.8%	43; 0.8%	31; 0.6%	27; 0.5%	26; 0.5%	25; 0.5%
Missing BUSCOs (M)	330; 6.3%	329; 6.2%	88; 1.6%	70; 1.3%	63; 1.2%	67; 1.3%
Total BUSCO searched	5286; 100%	5286; 100%	5286; 100%	5286; 100%	5286; 100%	5286; 100%

Assemblies (assembly name, species, BioProject, assembly accession): ilLegGlyc1.1 *Leguminivora glycinivorella*, PRJNA759210, GCA_023078275.1; ilCydSple1.1, *Cydia splendana* PRJEB45453 GCA_910591565.1; ilPamFasc1.1, *Pammene fasciana* PRJEB46632 GCA_911728535.1, ilNotUddm1.1, *Notocelia uddmanniana* PRJEB42037 GCA_905163555.1; ilApoTurb1.1, *Apotomis turbidana*, PRJEB41899 GCA_905147355.1

### Scaffolding

**Fig. 3:**
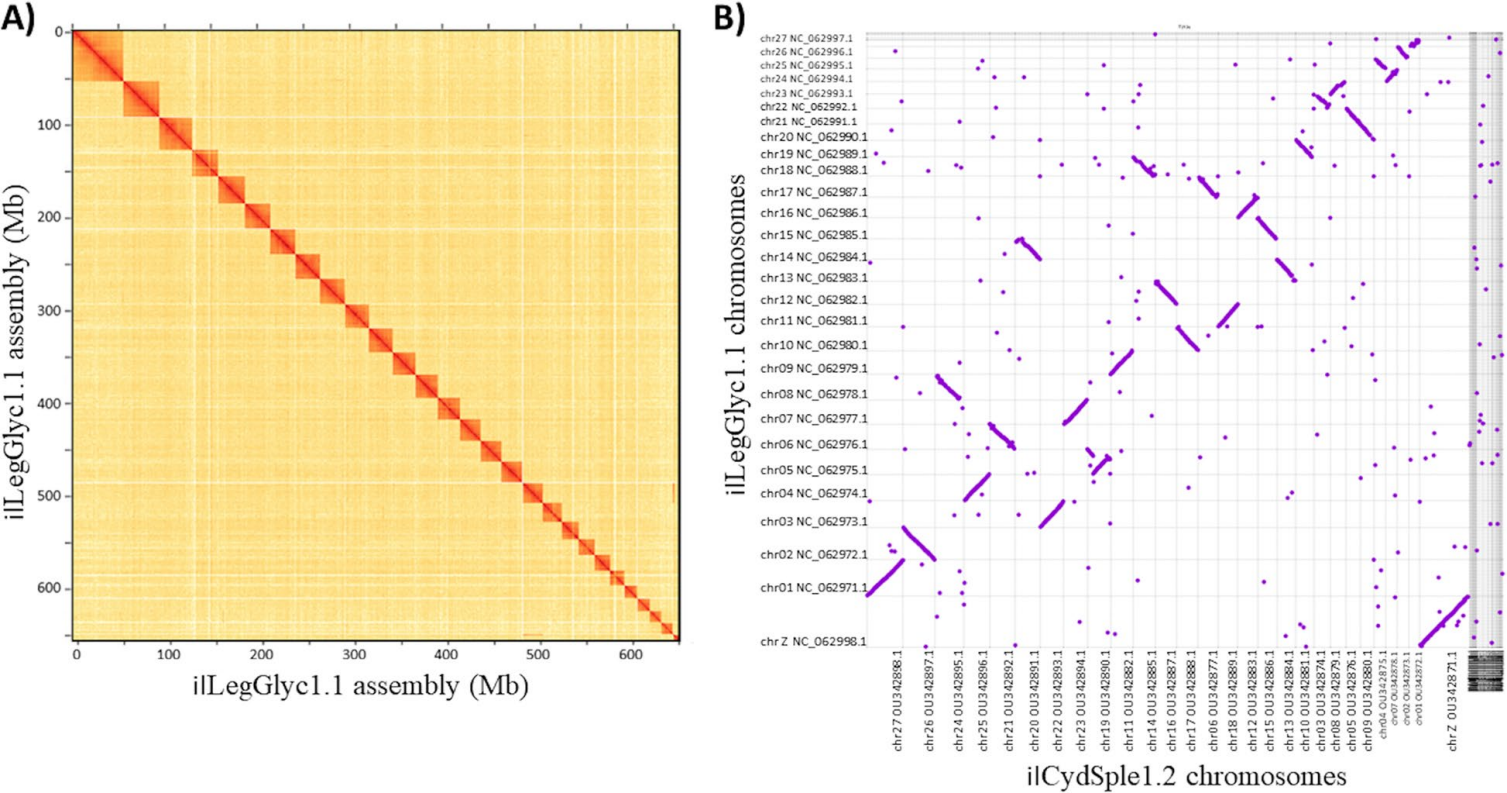
*Leguminivora glycinivorella* genome scaffolding and assignment to chromosomes. **A** chromosome conformation capture (Hi-C) contacts among 237 contigs defining 28 chromosomes in the *L glycinivorella* assembly ilLegGlyc1.1. **B** Whole genome alignment between ilLepGlyc1.1 and the *Cydia Splendana* assembly, ilCydSple1.2, defining putative orthologs and the Z chromosome (based on 5,625 alignments of 1711 ± 937 bp spanning at total of 0.8 Mb; Additional details in Table S3 and Figure S2)

Long range contacts predicted from Hi-C data aligned to the initial 225 contigs resulted in breaking of 11 misjoins in ilLegGlyc1.0. This refined set of 237 contigs were then joined into 40 scaffolds (NCBI accessions JAKXMO010000001-JAKXMO010000040) under the *L. glycinivorella* WGS Project JAKXMO01 of which 28 scaffolds were assigned to chromosomes (Chr; CM041121-CM041148; 27 autosomes + Z chromosome) and corresponding RefSeq chromosomes (NC_062971- NC_062998.1; Table S2; Fig. 3A). This chromosome-level scaffolded *L. glycinivorella* haploid assembly, ilLegGlyc1.1, had an N50 and longest scaffold (largest chromosome) of 25.3 Mb and 55.4 Mb, respectively (Table 1). Among genes in the BUSCO lepidoptera_odb10 set, scaffolds in ilLegGlyc1.1 contained 93.0% complete (91.5% S and 1.5% D) and 0.8% fragmented BUSCOs (F), while 6.2% were missing (M) (Table 2). BUSCO scores for ilLegGlyc1.1 were similar to those for assemblies from other Tortricid moths. This final scaffolded assembly, ilLegGlyc1.1, was submitted to DDBJ/ENA/GenBank under BioProject PRJNA759210 with assembly accession GCA_023078275.1 (Locus tag prefix: K7X69).

Whole genome alignment between scaffolded assemblies for *L. glycinivorella*, ilLegGlyc1.1 and *C. splendana*, ilCydSple1.2 contained 5,651 alignments (Table S3), of which 5,531 alignments had a one-to-one correspondence between chromosomes from the two assemblies (proportion ≥ 91.5% across chromosomes; range 91.5 to 100.0%) (Table S4). These alignments spanned a total of 9.75 Mb, with a range of 0.06 to 0.80 Mb between chromosomes, and corresponding mean identities and lengths of 1519.54 ± 613.66 bp and ≥ 87.90 ± 2.52%, respectively (Table S4). The number (count) of these one-to-one alignments were generally associated with corresponding chromosomal lengths, with exception of ilLepGlyc1.1 chr06 and 07 with ilCydSple1.2 chr05 and chr08, respectively (Figure S2). These results were used to propose putative orthology among 27 autosomes and the Z chromosome from assemblies ilLepGlyc1.1 and ilCydSple1.2 (Fig. 3B; Figure S2). Specifically, putative orthology between ilCydSple1.2 OU342871.1 (Z chromosome) and a single ilLegGlyc1.1 scaffold, NC_062998.1, was based on 467 alignments of 1711 ± 939 bp with 89.5 ± 3.0% identity spanning 0.8 Mb. This evidence was used to define NC_062998.1 as the Z chromosome in the *L. glycinivorella* Refseq assembly (GCF_023078275.1; scaffold accession JAKXMO010000028.1; chromosome accession: CM041148.1).

### Genome annotation

Based on ab initio and evidence-based predictions, 18,197 RefSeq gene models were constructed by the NCBI automated Eukaryotic Genomic Annotation Pipeline (Annotation Release 100; https://www.ncbi.nlm.nih.gov/genome/annotation_euk/Leguminivora_glycinivorella/100/; Table 1), among which 15,681 and 2,382 were protein coding and non-coding, respectively. The corresponding 26,672 RefSeq transcript models encoded 23,735 mRNAs (Table 1), with the remaining including long non-coding RNAs (lncRNAs; *n* = 1,042), tRNAs (*n* = 912), rRNAs (*n* = 75), snRNAs *n* = 51), and snoRNAs (*n* = 17). Among the 23,735 mRNAs, 21,155 (89.1%) were fully-supported by our RNA-seq evidence (Table S1; 485.3 million reads) and/or from read data generated in other experiments (BioSample IDs: SAMN07498241 and SAMN07498246 to SAMN0749824; 314.7 million reads). NCBI performed internal corrections of putative artifactual premature stop codons, frameshifts and insertion/deletions for 959 mRNAs, and 59 mRNAs remained as partial or incomplete sequences (remaining data not shown). WindowMasker predicted 48.7% of ilLepGlyc1.1 (assembly accession GCF_023078275.1) as repetitive DNA.

### Larval transcriptome response to feeding on transgenic *G. max* expressing Cry1Ac

**Fig. 4:**
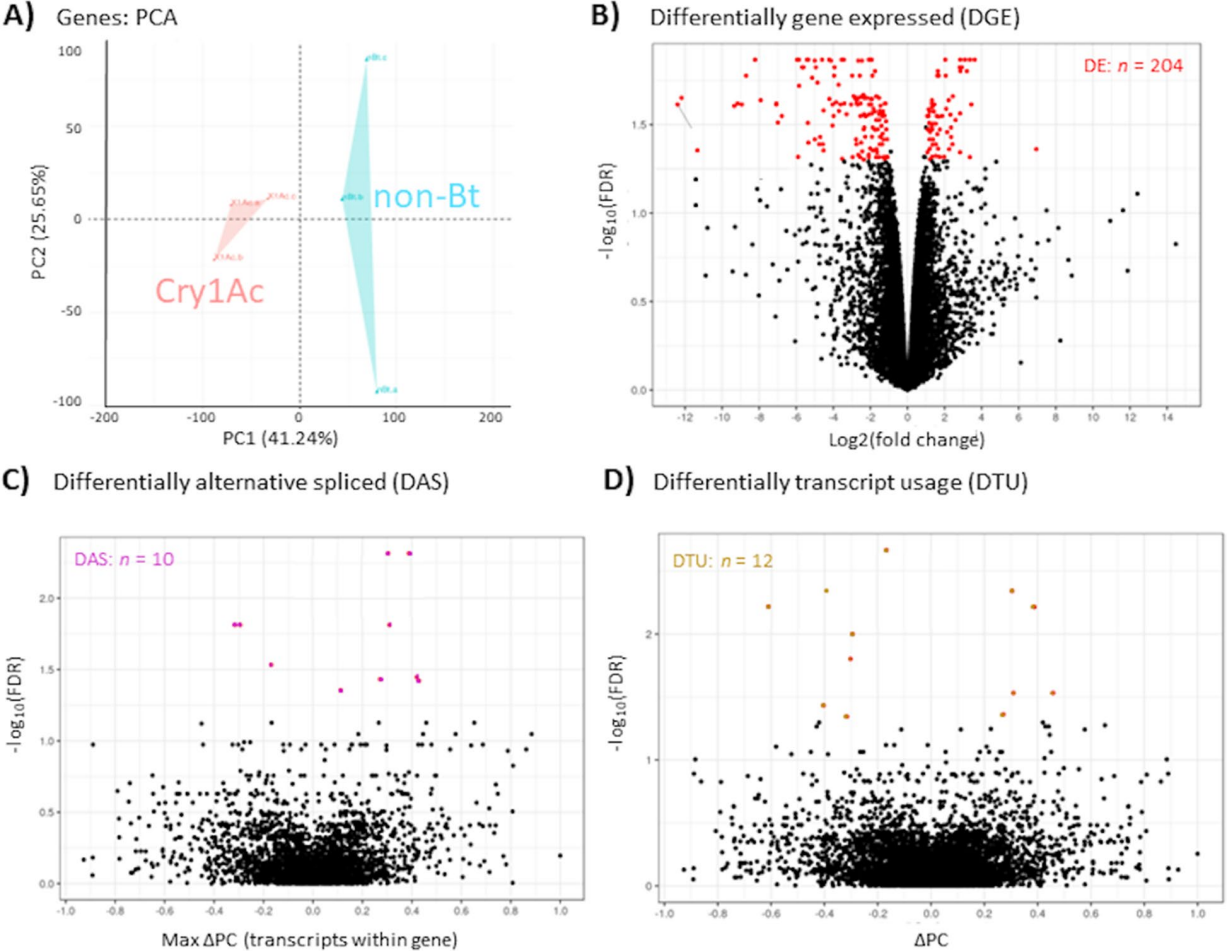
Effects of larval *Leguminivora glycinivorella* exposures to transgenic *Glycine max* cultivar GP03-8–23 expressing the *Bacillus thuringiensis* (Bt) Cry1Ac pesticidal protein compared to a non-Bt cultivar. **A** Principal component analysis (PCA) plot of mean log_2_CPM estimates among transcripts for genes along principal coordinates 1 (PC1) and 2 (PC2) showing two clusters corresponding to treatment groups. Variation between treatments precited **B** 204 genes with significant differential expression (DE) and **C** 10 genes showing significant differential alternative splicing (DAS). Similarly, at the transcript level **D** PCA demonstrate distinct clustering of read abundances among replicates of each treatment along PC1 and PC2. Intra-treatment comparisons predicted **E** DE of 39 transcripts and **F** 12 with predicted differential transcript usage (DTU)

**Fig. 5:**
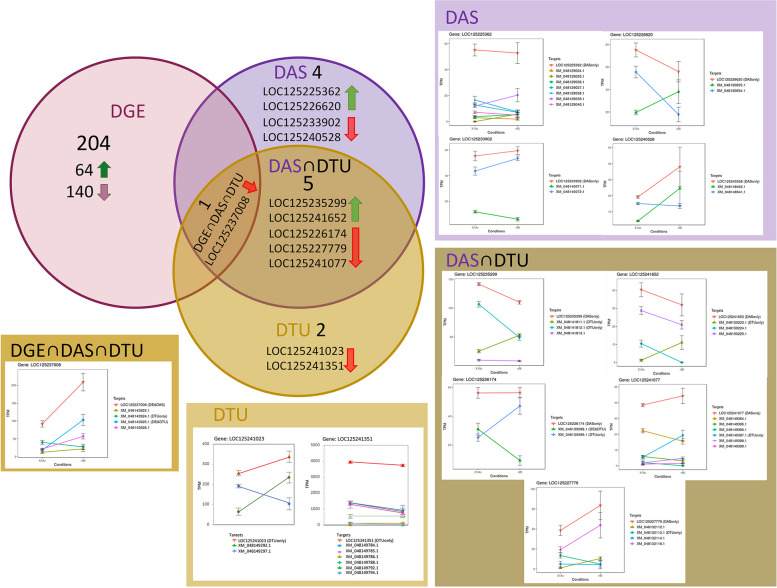
Differential expression between larval *Leguminivora glycinivorella* exposed to transgenic *Bacillus thuringiensis* (Bt) Cry1Ac *Glycine max* compared to a non-Bt cultivar. Significant changes between treatments defined for differential gene expression (DGE), differential alternate splicing (DAS) and differential transcript usage (DTU) as determined by 3D RNA-seq package v 1.0.0 (Guo et al. 2021). Absolute number of genes in each category are shown along with up- (  ) and down-regulated (  ) genes are given for DGE estimates, and individual genes for DAS, DTU and their intersections shown in the Venn-diagram. Transcript profile plots of length-scaled transcripts per million for each isoform between Cry1Ac and non-Bt exposure treatment conditions

Preprocessing filters removed transcript alignments with low read coverage and representation across replicates, and resulted in “smoothed” mean variance plots at gene (Figure S3A) and transcript levels (Figure S3B). Subsequent read counts normalized by log2(CPM) reduced inter-specific variance for the remaining 13,092 genes (Figure S4) and 18,561 transcripts (Figure S4B). A majority of the 13,092 gene models (*n* = 10,077; 77.0%) had single assigned transcripts. Principal Component Analysis (PCA) of mean log2(CPM) values, following correction for outlier effects, showed intraspecific clustering of replicates within Cry1Ac and non-Bt treatments at the gene level (Fig. 4A), where PC1 and PC2 accounted for 41.24 and 25.62% of the variance, respectively. From these estimates of read abundances among the 13,092 filtered gene set, there was a range of -12.40 ≥ Log2(fold-change) ≤ 14.47 between larval *L*. *glycinivorella* Cry1Ac and non-Bt treatment groups (Table S5a). A total of 204 genes were predicted to show significant DGE (Fig. 4B; Table S5b), and 10 genes to have significant DAS (Fig. 4C; Table S5c). Among the significant DGE predictions, 64 and 140 were up- and down-regulated (Fig. 5), respectively. The gene LOC125237008, a putative GTP cyclohydrolase, with significant DGE was also predicted to have significant DAS between encoded transcripts XM_048143924.1 and XM_048143925.1. Additionally, 12 significant DTU events were predicted (Table S5d), which by nature of their definitions, encompassed transcript isoforms assigned to the 10 genes with significant DAS (Table S5e; Fig. 5). Transcripts from 5 genes were predicted to show both DAS and DTU. DAS between transcripts XM_048143924.1 and XM_048143925.1 from LOC125237008 was based on DTU, and only predicted with DGE, DAS and DTU. Furthermore, transcripts XM_048149292.1 and XM_048149297.1 from LOC12524102, a probable mitochondrial zinc-binding oxidoreductase, and transcript XM_048149794.1 from LOC125241351 encoding a putative metalloprotease tolloid (TLD) domain-containing protein showed only significant DTU compared to other transcripts derived from respective loci.

**Fig. 6:**
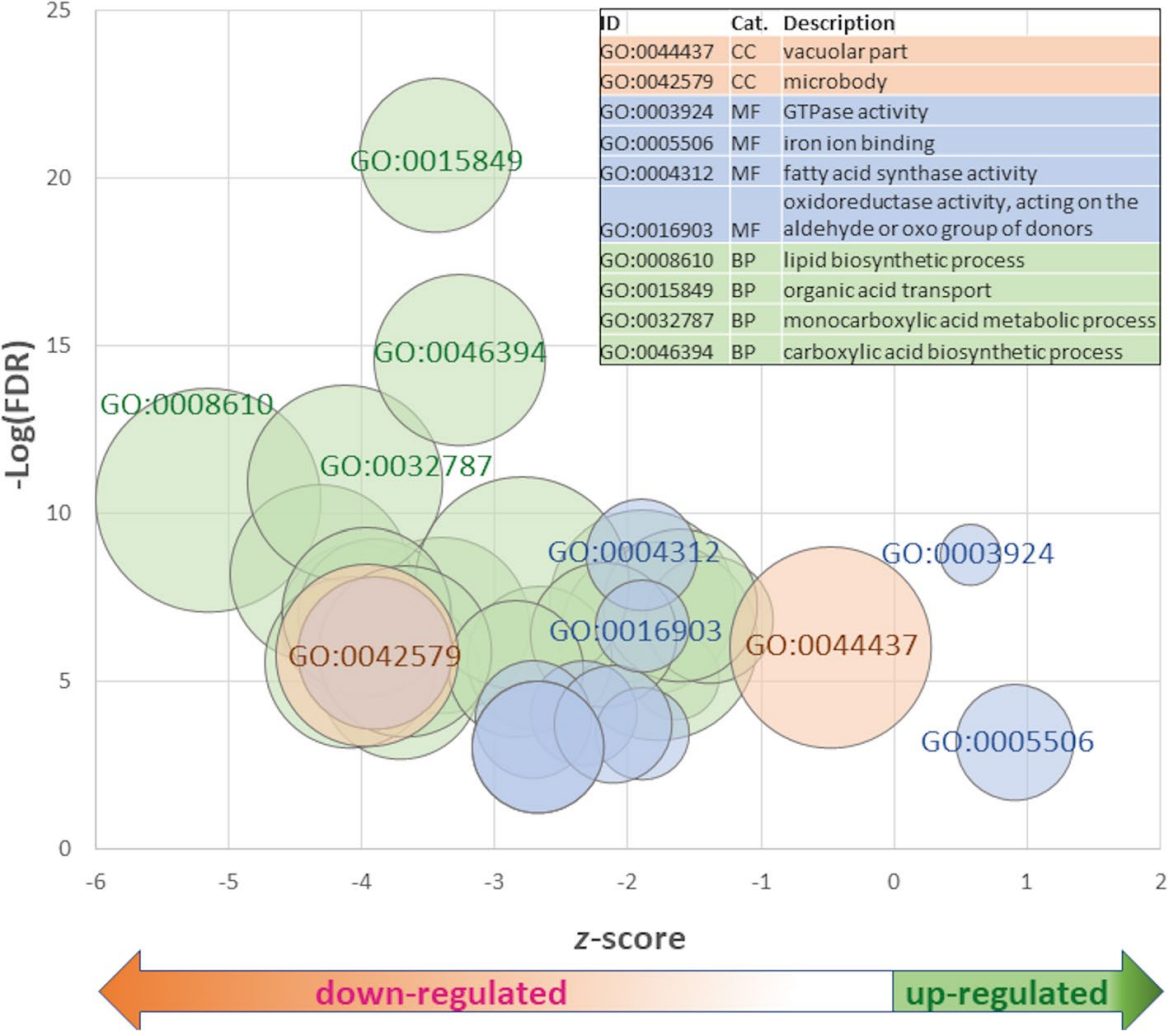
Significantly enriched Gene Ontologies (GOs) in cellular component (CC), biological process (BP) and molecular function (MF) categories at GO level 2. Z-score indicative of genes in category being more up- (positive) or down-regulated (negative value). Top four most significantly enriched GOs or GOs with greatest expression bias in each category demarcated

There were 104 unique Pfam domains predicted in the 204 (PfamScan* E*-values ≤ 1.0 × 10^–11^; remaining data not shown), from which 203 associated curated GO terms were retrieved across CC, BP and MF categories from level 1 to 4 (Table S6). These encompassed 2, 22, and 12 CC, BP and MF categories at GO level 2, respectively (Table 3). The most significantly enriched terms for CC and BP categories were vacuolar part and microbody, and secondary metabolic and organic hydroxy compound metabolic processes, respectively. Most significant enrichment was predicted for fatty acid synthase activity and oxidoreductase activities among MF terms. Simultaneous assessment of significance, directional bias of DEGs (up- or down-regulated), and number of DEGs in each enriched GO category (Fig. 6) showed that BP GO:0008610 (lipid biosynthetic process), GO:0015849 (organic transport), GO:0032787 (monocarboxylic acid metabolism), and GO:0046394 (carboxylic acid biosynthesis) are most significantly enriched and represent groups of genes biased for reduced expression. The MF terms GO:0003924 (GTPase activity) and GO:0005506 (iron binding) contained genes with a positive (increased) expression bias, whereas DEGs in GO:0004312 (fatty acid synthase activity) and GO:0016903 (oxidoreductase activity) were mostly down-regulated in Cry1Ac exposed larvae. Six genes in enriched GO categories are putatively in cytochrome P450 superfamilies, all of which were significantly down-regulated. Genes with putative function in chitin maintenance were significantly up-regulated [chitinase (LOC125235932, LOC125235952, and LOC125235918 Table S7a-c); and deacetylase activities (LOC125236753)]. Terms for DEGs encoding constituents of the peritrophic membrane and cuticle [peritrophin-1-like (LOC125238640); and cuticle protein LCP-17-like (LOC125230076)], although their common MF term, chitin binding (GO:0008061)] were not among those that were significantly enriched.

**Table 3:** Significantly enriched Gene Ontologies (GOs) assigned to genes differentially expressed between 2nd instar *Leguminivora glycinivorella* larvae fed on Cry1Ac compared to non-Bt *Glycine max* among cellular component (CC), biological process (BP) and molecular function (MF) categories at GO level 2. False discovery rate (FDR) thresholds of 1.0^–4^ applied CC and MF categories, and ≤ 1.0^–6^ to category terms

Cat	GO ID	GO term	level	Z-score	FDR
CC	GO:0044437	vacuolar part	2	7.90	9.79E-07
CC	GO:0042579	microbody	2	7.76	1.74E-06
MF	GO:0004312	fatty acid synthase activity	2	12.8	1.71E-09
MF	GO:0016903	oxidoreductase activity, acting on the aldehyde or oxo group of donors	2	9.77	2.26E-07
MF	GO:0005342	organic acid transmembrane transporter activity	2	8.40	1.41E-06
MF	GO:0008509	anion transmembrane transporter activity	2	6.40	3.32E-05
MF	GO:0050662	coenzyme binding	2	6.17	3.69E-05
MF	GO:0005319	lipid transporter activity	2	6.83	8.45E-05
MF	GO:0005506	iron ion binding	2	6.42	1.37E-04
MF	GO:0046906	tetrapyrrole binding	2	6.18	1.89E-04
MF	GO:0015238	drug transmembrane transporter activity	2	5.98	3.50E-04
MF	GO:0003924	GTPase activity	2	5.72	5.11E-04
MF	GO:0016247	channel regulator activity	2	5.48	6.50E-04
MF	GO:0016705	oxidoreductase activity, acting on paired donors, with incorporation or reduction of molecular oxygen	2	4.97	9.35E-04
BP	GO:0019748	secondary metabolic process	2	18.43	2.12E-21
BP	GO:1,901,615	organic hydroxy compound metabolic process	2	11.68	7.11E-13
BP	GO:0032787	monocarboxylic acid metabolic process	2	10.68	1.24E-11
BP	GO:0008610	lipid biosynthetic process	2	10.24	3.95E-11
BP	GO:0009410	response to xenobiotic stimulus	2	10.85	6.30E-09
BP	GO:0033993	response to lipid	2	9.00	1.22E-08
BP	GO:0010817	regulation of hormone levels	2	8.76	4.53E-08
BP	GO:0032870	cellular response to hormone stimulus	2	8.66	5.51E-08
BP	GO:0055114	oxidation–reduction process	2	7.91	8.37E-08
BP	GO:0015849	organic acid transport	2	9.20	1.01E-07
BP	GO:0021700	developmental maturation	2	9.03	1.40E-07
BP	GO:0006820	anion transport	2	8.29	1.62E-07
BP	GO:0001101	response to acid chemical	2	8.33	2.12E-07
BP	GO:0062012	regulation of small molecule metabolic process	2	8.17	4.16E-07
BP	GO:0017144	drug metabolic process	2	6.93	6.27E-07
BP	GO:0016319	mushroom body development	2	10.59	9.11E-07
BP	GO:1,901,701	cellular response to oxygen-containing compound	2	6.97	1.28E-06
BP	GO:0019216	regulation of lipid metabolic process	2	7.54	2.14E-06
BP	GO:0046394	carboxylic acid biosynthetic process	2	6.67	2.71E-06
BP	GO:1,905,952	regulation of lipid localization	2	8.20	4.27E-06
BP	GO:0016198	axon choice point recognition	2	9.64	7.12E-06
BP	GO:0006790	sulfur compound metabolic process	2	6.60	8.01E-06

Additionally, some genes previously identified as involved in Bt resistance among species of Lepidoptera showed significant DGE (Table 4; Table S5b; Table S7a-c). These included two ABC transporters in subfamily C and G members and 4 tetraspanin orthologs (Table 4), all of which were assigned to significantly enriched GO categories. Significant DGE was also predicted for an up-regulated membrane-bound aminopeptidase N (*apn*) and down-regulated alkaline phosphatase (*alp*) orthologs. The *L*. *glycinivorella* gene, LOC125230228, encoding two RefSeq annotated *alp* transcript isoforms, XM_048135319.1 and XM_048135320.1, was significantly down-regulated ion in Cry1Ac exposed larvae (Log2FC = -2.10; FDR = 0.0136; Table S5a). An amino acid sequence alignment with *Heliothis virescens* mALPs (HvmALP) predicted* L*. *glycinivorella* mALP protein XP_047991276.1 (isoform X1) encoded by XM_048135319.1 and XP_047991277.1 (isoform X2) encoded by XM_048135340.1, had 48.12 and 46.99 percent identity with HvmALP1 and HvmALP2, respectively (remaining results not shown).

**Table 4:** Candidate proteins involved in *Bacillus thuringiensis* (Bt) insecticidal resistance and detoxification enzyme encoded by differentially-expressed genes (DEGs) between susceptible 2nd instar *Leguminivora glycinivorella* larvae fed on transgenic Cry1Ac *Glycine max* compared to unexposed cohorts

A) DEGs significant between treatments			Pfam	Putative	Log_2_
Locus	Identifier	GO ID (GO categories)	domains	RefSeq gene description	(FC)
LOC125238640	XP_48001964.1	GO:0008061 (MF); GO:0005576 (CC)	PF01607	peritrophin-1-like	+ 2.22
LOC125242528	XP_048007237.1 XP_048007238.1	GO:0008237 (MF); GO:0008270 (MF)	PF17900PF01433 PF11838	aminopeptidase N-like	+ 2.06
LOC125230228	XP_047991277.1 XP_047991276.1	GO:0016791 (MF)	PF00245	membrane-bound alkaline phosphatase-like	-2.09
**B**) DEGs significant between treatments and in significantly enrich GO terms			Pfam	Putative	
Locus	Proteins(s)	GO ID (GO categories)	domains	RefSeq gene description	Reg
LOC125235293	XP_047997762.1	GO:0044437 (CC); GO:0021700 (BP)	PF00335	Tetraspanin	+ 1.68
LOC125229025	XP_047989739.1			Tetraspanin	+ 1.18
LOC125235440	XP_047997964.1			Tetraspanin	+ 1.14
LOC125236892	XP_047999767.1			Tetraspanin-12	-1.49
LOC125238198	XP_048001427.1	GO:0042579 (CC); GO:0008509 (MF); GO:0005342 (MF); GO:0016247 (MF); GO:0015238 (MF); GO:0005319 (MF); GO:0001101 (BP); GO:0006820 (BP); GO:0009410 (BP); GO:0015849 (BP); GO:0019748 (BP); GO:0021700 (BP); GO:0032787 (BP); GO:0032870 (BP); GO:0033993 (BP); GO:0055114 (BP); GO:1,901,701 (BP); GO:1,905,952 (BP)	PF00005 PF01061	ABC transporter subfamily G (ABCG)	-1.50
LOC125240824	XP_048004907.1		PF00005 PF00664	ABC transporter subfamily C4 (ABCC4)	-2.50
LOC125235547	XP_047995568.1	GO:0005506 (MF); GO:0016705 (MF); GO:0046906 (MF); GO:0006790 (BP); GO:0008610 (BP); GO:0009410 (BP); GO:0010817 (BP); GO:0017144 (BP); GO:0019216 (BP); GO:0019748 (BP); GO:0033993 (BP); GO:0046394 (BP); GO:0055114 (BP); GO:0062012 (BP); GO:1,901,615 (BP); GO:1,901,701 (BP); GO:1,905,952 (BP); GO:0032787 (BP)	PF00067	Cytochrome P450s	-1.16
LOC125228160	XP_047988575.1				-2.20
LOC125242592	XP_048007337.1				-2.33
LOC125238349	XP_048001606.1				-3.40
LOC125235906	XP_047998497.1				-3.93

A) significantly DEGs between treatments, and B) DEGs annotated with Gene Ontology (GO) terms that were significantly enriched. Putative functional annotations provided for GO cellular component (CC), biological process (BP) and molecular function (MF) categories at GO level 2, with Pfam domain predictions and RefSeq gene descriptions indicated. Direction and magnitude of estimated Log_2_(fold-change) [Log_2_(FC)] among replicated treatment groups is shown for each significant DEG (false discovery rate ≤ 0.05)

Phylogenetic reconstruction of the aminopeptidase N gene family among orthologs from *B. mori*, *L. glycinivorella*, and *O. nubilalis* supported 18 putative clades, of which 16 corresponded to those in *B. mori* (Figure S5). The two additional clusters corresponded to putative *L. glycinivorella*-specific APN03- and APN07-like clades. The APN07-like clade contained aminopeptide N-like proteins XP_48003237.1 and XP_48003238.1 from LOC125242528, where LOC125242528 was significantly up-regulated in Cry1Ac exposed *L. glycinivorella* larvae (Log2FC = 2.06; FDR = 0.0167; Table S5a).

## Discussion

### Chromosome-level genome resources

Investigation of Bt intoxication in this study was facilitated by *L. glycinivorella* genomic resources comprised of a structurally annotated chromosome-level genome assembly. The 27 autosomes and a Z-chromosome in the scaffolded assembly, ilLegGlyc1.1, is expected based on karyotype data suggesting the ancestral chromosome number (N) among Lepidoptera in the Family Tortricidae, N = 30. This chromosome number is reduced to 28 in Subfamily Olethreutinae [68] to which *L. glycinivorella* belongs (Taxomony ID 10351111). Due to the ZW/ZZ female/male sex determination system of Lepidoptera, the homogametic male assembly ilLegGlyc1.1 lacks a W-chromosome, as does assemblies from other tortricids (Table 2). Although not investigated here, the Z-chromosome of tortricid moths is of interest due to predicted ancestral autosomal fusion [69], and involvement in speciation [70] and ecotype variation among Lepidoptera [71]. Nearly 90% of the 23,735 NCBI RefSeq gene annotations for ilLegGlyc1.1 are supported by transcript evidence (Table 1), and provides a resource for current and future research.

### Transcriptional responses to transgenic Bt pesticidal plant exposure

The development of phenotypes in insect populations that are resistant to Bt insecticidal proteins reduce the efficacy of control strategies and pose a threat to the sustainability of current agricultural production practices. Mechanism(s) of lepidopteran resistance to Bt crystalline (Cry) insecticidal proteins are reported to involve structural or functional changes to the proteins that mediate binding and pore formation in the gut, alter proteolytic cleavage of native Bt toxins, or increased immune, cellular regeneration, and toxin sequestration capacities [30, 72–75]. Additionally, intracellular protein kinase signaling pathways are involved in Bt MoA [25] and are altered in resistance insects [34]. The role of mutations in genes tetraspanin [76] and kinesin [77] in Bt Cry1Ac toxin resistant *Helicoverpa* sp. and MoA of Bt intoxication remain enigmatic. Even though Bt Cry1A proteins may interact with different midgut receptor proteins compared to Cry1F [78], and the Bt vegetative insecticidal protein, Vip3, interacts with a different set of midgut receptors compared to Cry1 proteins [79, 80], a meta-analysis provided evidence for weak cross resistance between Cry1 and Vip3A [81]. These prior studies suggest that Bt MOA and points wherein disruption(s) lead to resistance are not yet fully understood, which could be assisted by further investigation of molecular and cellular impacts of Bt intoxication [37, 39]. Specifically, this would involve expanding knowledge of the molecular interactions and pathways that invoke cell death or recovery and repair following Bt intoxication, which ultimately determine organismal survival or mortality, and be informed by molecular responses of susceptible insects when exposed to Bt proteins.

In our study, relatively few genes (*n* = 204) were predicted to be significantly differentially expressed between *L. glycinivorella* larvae feeding on Cry1Ac (cultivar GP03-8–23) and a non-Bt cultivar compared to analogous results from prior studies involving species of Lepidoptera. Specifically, prior analyses predicted thousands of DEGs between Bt and non-Bt exposed lepidopteran larvae [41–43, 47]. Also, despite 104 of these 204 predicted DEGs being significantly down-regulated in Cry1Ac exposed *L. glycinivorella* (68.6%), there was a bias for down-regulation among genes assigned to significantly enriched GO categories (Fig. 6; 89.4% (17 of 19), 78.3 (54 of 69), 78.8% (78 of 99) in enriched CC, MF, and BP categories, respectively). Only exceptions were genes in MF categories GO:0003924 (GTPase activity) and GO:0016247 (iron ion binding) that were weakly biased for up-regulation. Although not investigated further, differences with prior studies in Lepidoptera may be influenced by different larval exposure times, level of exposure (dose), or other unaccounted for factor(s). Due to use of on-plant treatments (exposures) in our study, the absolute dose remains unknown compared to prior reports where exposures were to known concentrations of purified Bt protein in artificial diet bioassays [41–43, 47]. Regardless, clustering of PCA of Log_2_CPM estimates suggests relative responses that are homogeneous within and heterogeneous between *L. glycinivorella* larval treatment groups (Fig. 4A), indicating on-plant assays invoke a reproducible molecular response. It remains possible that transcriptional responses to undescribed differences in host plant resistance (HPR) factors between Bt and conventional cultivars may overlap with responses to Bt Cry1Ac exposures or be induced in both our treatments. This confounding factor could relegate a portion of genes involved in general response to Bt and unrelated HRP traits as non-significant and may be partially explanatory of the relatively small number of DEGs predicted in this study.

### Changes in pathways associated with cell stress responses

Our results provide insights into the effects of feeding on transgenic Bt crops on gene expression by a susceptible pest insect and suggest a set of putative genes and pathways may be involved in physiological responses. Although functional GO annotations are extrapolated from model organisms, they are yet to be validated in *L. glycinivorella.* Enriched CC, BP and MF categories offer a baseline for interpreting potential systemic effects of feeding by susceptible insects on Bt compared to non-Bt plants. For instance, the MF category oxidoreductase activity was the second most significantly enriched GO category among *L. glycinivorella* DEGs (Table 3), agreeing with prior evidence that pathways which remediate oxidative stress are affected by Bt intoxication [82]. Genes encoding cytochrome P450 monooxygenases were down-regulated in Cry1Ac exposed *L. glycinivorella*, suggesting further cellular efforts to reduce the accumulation of reactive oxygen species (ROS). A number of BP categories for metabolic processes are modulated after Cry1Ac exposure. Metabolic changes were also predicted in response to oxidative stress in *H. armigera* [83], and more generally following exposures to insecticidal toxins [39, 84, 85]. Cellular repair and survival mechanisms are also accompanied by increased metabolic rates [86, 87] and are mechanisms by which Cry1Ac resistant *H. armigera* respond to Cry1Ac exposure [47]. These lines of evidence may support a hypothesis that changes in metabolic processes predicted in this study could be a consequence of or in support of stress response and repair mechanisms induced by Cry1Ac exposure in susceptible *L. glycinivorella* larvae.

The two enriched CC categories in our study, vacuole part and microbody, interestingly were also significantly enriched among genes down-regulated in larvae of the coleopteran *D. v. virgifera* following exposures to transgenic Bt Cry3Bb1- and Tpp34/Gpp35Ab1 maize [39]. This commonality could point to involvement of recycling damaged cell components and adaptation to stress in Bt exposure responses. Specifically, microbodies were described as a suite of single membrane-enclosed organelles [88] that encompass lysosomes and peroxisomes [89]. Peroxisomes are involved in lipid biosynthesis and house oxidative reactions that form peroxides [90], and are self-replicating and provisioned by importation of enzymes. Lysosomes are endosomal vesicles produced by the *trans*-Golgi network that function in autolysis of intracellular components and houses machinery involved in autophagy, a conserved eukaryotic mechanism that degrades and recycles damaged cellular components to maintain homeostasis [91]. For terminology’s sake, the vacuole (yeast and plants) is synonymous with the lysosome (animalia) [92]. Aspects of cell stress are mediated by functions of lysosomes [93] and peroxisomes [94], which may include autophagy as a response to Bt infection or toxin exposure [95, 96].

Other studies implicate an apoptotic response to Bt toxins [39, 82, 97, 98]. Similar to a prior study in *D. v. virgifera* [39], a putative lifeguard-1-like protein (LG1) with a B-cell-lymphoma protein 2 (Bcl-2)-associated X (BAX) inhibitor (Bax1-I) domain was significantly up-regulated in *L. glycinivorella* response to Bt exposure. Bax1-I domain proteins are involved in the negative regulation of apoptotic signaling pathways through the remediation of cell stress, where lifeguard does so by reduction of stress in the Golgi and endoplasmic reticulum [99]. In our study, LG1 is encoded by *L. glycinivorella* LOC125231679 and is significantly up-regulated following Cry1Ac exposure. When considered in conjunction with modulation of pathways that remediate damage by ROS, LG1-mediated suppression of apoptosis may indicate survival mechanisms are engaged following Bt-induced cellular damage [39], and similarly occur among Cry1Ac-exposed *L. glycinivorella* larvae. Granted these general conclusions are drawn upon comparisons among a relatively limited set of experiments, but meta-analyses of these and future studies may shed light on conserved underlying pathways involved in Bt intoxication and potentially inform steps whereby resistance might develop.

### Differential-regulation of putative Bt binding protein coding genes

Proteins in the midgut of Lepidoptera function as receptors in a sequential binding model for pore-forming Cry1A pesticidal proteins [23, 24, 100]. Interestingly, genes encoding orthologs of some of these receptors show significant differential expression between susceptible *L. glycinivorella* fed on Cry1Ac transgenic *G. max* compared to those fed on a non-Bt cultivar (Table 4). It should be cautioned that changes in transcript levels are not necessarily reflected in corresponding protein levels, and latter have not been validated. Regardless, two transcripts encoding putative glycosylphosphatidylinositol (GPI)-anchored membrane-bound Bt receptor proteins, alkaline phosphatase (mALP) and aminopeptidase (APN), are differentially regulated. Specifically, we predicted that a *L*. *glycinivorella malp* is significantly down-regulated in Cry1Ac exposed larvae (Table S5a), and that two RefSeq protein isoforms encoded by *L*. *glycinivorella malp* are orthologous to *H. virescens* HvmALP1 and HvmALP2 that show a strong correlation between reduced transcript and protein levels in Cry1Ac resistant strains of *H. virescens* [101]. Additional evidence shows that mALP is a functional receptor for Cry1A and Cry2Ab2 proteins [102], but not Vip3A [103], where N-acetylgalactosamine modification is required for Cry1Ac binding [78]. Furthermore, constitutively reduced mALP or *malp* levels are documented in Cry1Ac resistant laboratory strains of *Helicoverpa armigera* and *Spodoptera frugiperda* [101], Cry1Ab resistant *Ostrinia nubilalis* [43] and Cry1C resistant *Chilo suppressalis* [104], but not in Cry1Ab resistant *Diatraea saccharalis* [96]. Ours is the first known report of induced *malp* down-regulation following Cry protein exposure among Lepidoptera, but expression of several *alp* gene family members decreased in *Aedes aegypti* following intoxication with insecticidal Bt subsp. *israelensis* [105]. The role or consequence of these changes following Bt intoxication in *L*. *glycinivorella* and other insects remains unknown.

Our results also predicted the significant up-regulation of gene LOC125242528 in Cry1Ac cultivar exposed larvae (Table 4), which encodes a membrane-bound alanyl aminopeptidase (APN). Phylogenetic reconstructions among members of this gene family in lepidopteran species predict 8 [106] or 16 clades [107]. Our corresponding interspecific phylogenetic reconstruction predicts 16 major clades corresponding to those in *B. mori*, wherein *L. glycinivorella* gene expansion is most abundant in an APN05 clade with three paralogs as compared to one in each *B. mori* [108] and *O. nubilalis* [109] (Figure S5). Protein products from LOC125242528 cluster adjacent to members of an APN07 clade that also includes products from a *L. glycinivorella* APN-encoding gene, LOC125242529. Our phylogenetic analysis also indicates that the up-regulated *L. glycinivorella* APN7-like gene LOC125242528 is not orthologous with APN1 and APN3, where one or both *apn*1 and *apn*3 are down-regulated in Cry1A resistant *Helicoverpa armigera* [110], *Trichoplusia ni* [111], *O. nubilalis* [112], *O. furnacalis* [44], and Cry1C resistant *S. exigua* [113]. Although some APN orthologs are functional Cry1A receptors [114], confer resistance in knock-out strains of *Plutella xylostella* [115], and interact with oligomerized Bt protein pre-pore structures to facilitate membrane insertion [23, 24], APNs have a diversity of physiological roles or are not expressed in gut tissues [107]. Specifically, *apn*1, 2, 4, 5, 6, 9, and 12 are expressed in midgut of larval *B. mori*, and all of these but *apn*12 are up-regulated at 6 h post-infection with a strain of *Bacillus bombyseptieus* [107]. Initial *apn* transcript up-regulation similarly occurred in analogous experiments, but significantly down-regulated by 24-h post *B. bombyseptieus* infection [116]. Our study shows a putative *L. glycinivorella* APN07-like transcript being significantly up-regulated 48-h post exposure to Cry1Ac-expressing *G. max*. Since there is no evidence of this APN07-like protein or orthologs functioning as Bt receptors, observed changes could be indicative of proteolytic changes involved in other cellular processes [117, 118]. Although not investigated further, differences in expression among APN gene family members following Bt exposure may reside in differences in exposures times and method of protein exposure (bacteria, transgenic plant, or artificial diet incorporated).

We predicted that the expression of an ABC transporter from subfamily C (ABCC) was reduced in Cry1Ac *G. max* exposed *L. glycinivorella* larvae (Table 4). ABC transporters are implicated in Bt resistance following discovery of an insertional knockout of ABCC2 linked to Cry1Ac resistance in *H. armigera* [119] and subsequent associations shown in other species [30, 120]. Interestingly, down-regulation of *abcc*2 and *abcc*3 was associated with Cry1Ac resistance in *Plutella xylostella* [121], where these paralogs show functional redundancy as Bt receptors in *H. armigera* [122]. Despite this evidence, the ABCC subfamily member down-regulated in *L. glycinivorella* Cry1Ac-exposed larvae was assigned as a member 4 ortholog (ABCC4), which has no evidence as being a functional Cry1Ac receptor among species of Lepidoptera. Regardless, ABC transporters function the eflux of various cellular substrates [123] including metabolites from xenobiotic breakdown in insects [28], and are involved in mammalian stress-response pathways [124]. The down-regulation of ABCC4 in *L. glycinivorella* Cry1Ac-exposed larvae remains perplexing, but was not investigated further in this study.

Tetraspanin paralogs are significantly up- (*n* = 3) or down-regulated (*n* = 1) in Cry1Ac-exposed *L. glycinivorella* compared to unexposed cohorts (Table 4). Tetraspanins are an evolutionarily conserved gene family of proteins structurally composed of four transmembrane domains, and intra- and extracellular domains [125]. Binding of different ligands to a unique extracellular domain of each paralog transduce signals responsible for cellular responses such as migration, adhesion, and intracellular trafficking [88]. There are 36 paralogs in the *D. melanogaster* genome [126], and functional redundancies among paralogs have made it difficult to resolve independent roles in cellular processes [125, 127]. Mutational change at an amino acid position (L31S) in the first transmembrane domain of an *H. armigera* tetraspanin, HaTSPAN1, is linked to Cry1Ac resistance, and although HaTSPAN1 transcript levels are also reduced 2.7-fold there is no alteration in Cry1Ac binding [76]. Transcripts encoding tetraspanin orthologs were also up-regulated among susceptible *D. v. virgifera* larvae feeding on transgenic Cry3Bb1 and Gpp34/Tpp35Ab1 maize [39]. Although members of the tetraspanin gene family are involved in insect immune response [128] and recent evidence of differential regulation of immunity genes in Cry1Ac exposed strains of *H*. *armigera* [47], there is no analogous evidence from our study to suggest tetraspanin effects on immunity (Table 3; Table S5b). Thus, the role of tetraspanins in larval *L. glycinivorella* response to transgenic Cry1Ac *G. max* remains unknown.

It should be noted that there is a functional difference between Bt binding proteins and Bt receptor, where the latter is defined as proteins that facilitate membrane insertion [129]. Within this framework, ABC transporters and cadherin are purported to be major and “accessory’ functional receptors, respectively. Thus, changes in these two receptors my incur changes in resistance. Despite this, there instances where resistance has evolved outside of modulation of known receptor interactions [30, 76, 77], suggesting that gains in understanding of the Bt MoA might facilitate deciphering those mechanisms involved.

## Materials and methods

### Genomic library construction, sequencing, and read filtering

A hybrid short- and long-read approach was used to assemble *L. glycinivorella* contigs. For this, greater than 20 live 4th instar larvae were collected from a cultivated non-Bt *G. max* field at the Jilin Academy of Agricultural Sciences, Northeast Agricultural Research Center (JAAS-NARC), Gongzhuling, Jilin Province, P.R. China on 20 August 2020 (BioSample SAMN21160035; Isolate SPB_JAAS2020; Table S1). Samples were brought to the lab and frozen alive in a -80 ℃ freezer. A subset of samples then shipped on dry ice to Benagen Tech Solutions Ltd. (Wuhan, China) where genomic libraries were constructed and sequenced. In brief, total genomic DNA was extracted from a single male *L. glycinivorella* larva (larva #1; Table S1) using an in-house modified CTAB protocol, quality checked by 0.75% agarose gel electrophoresis, and quantified on a Qubit 3.0 Fluorometer (Life Technologies, Carlsbad, CA, USA). Genomic DNA was selected for fragments > 15 kb using AMPure XP beads (Beckman-Coulter Life Sciences, Indianapolis, IN, USA), repaired using the NEBNext FFPE DNA Repair Mix (New England Biolabs, Ipswich, MA, USA), and used as input for the SQK-LSK109 Ligation Sequencing Kit according to manufacturer instructions (Oxford Nanopore, Oxford, UK). Following quantification and purification, the large-insert library was loaded into R9.4 Spot-On Flow Cells and run on a PromethION P48 Sequencer (Oxford Nanopore). GUPPY v 4.0.2 (Oxford Nanopore) was used to convert image files to base calls and remove failed reads from the raw data, then trim reads of adapters and those with a Phred quality score (*q*) < 7.

Remaining DNA (< 15 kb) from *L. glycinivorella* larva #1 (Table S1) was sheared and fragments used to construct an indexed short insert library with the Nextera DNA Flex Library Prep Kit according to manufacturer instructions (Illumina, San Diego, CA, USA). Library quality was assessed on an Agilent 2100 Bioanalyzer Instrument (Agilent Technologies, Santa Clara, CA, USA), and quantitative PCR (qPCR) was used to estimate effective concentration as described previously [130]. Then 150 bp paired end (PE) reads were generated from the short-insert library on a single S4 flow cell lane of a NovaSeq 6000 (Illumina). Fastq formatted short read data was trimmed of adapters and sequence with low quality (*q* < 5), and PCR duplicates removed using in-house scripts at Benagen Tech Solutions Ltd.

### K-mer based genome size estimates

A kmer depth-frequency distribution was generated for short Illumina reads using kmer_freq from which the mean heterozygosity, repetitive fraction, duplication level, and genome size were estimated using the Genome Character Estimator (GCE) v.1.0.0 (https://github.com/fanagislab/GCE) [131] with default settings. This k-mer distribution was used as input to estimate genome size using the Lander-Waterman equation [132] and other parameters the R script GenomeScope2 [133].

### Contig assembly

The string graph-based de novo assembler for long reads, NextDeNovo v.2.5.0 (GrandOmics, Beijing, China), that applies an initial internal error correction of long reads prior to assembly, was used with default settings to generate a primary contig assembly. The resulting contigs were subjected to two successive rounds of error correction (e.g. “polishing”) with short Illumina PE read data (Table S1; post-filtered) using default settings of Racon v.1.4.11 (https://github.com/lbcb-sci/racon), followed by two additional rounds of polishing using Pilon v.1.23 [134]. The final draft contig assembly was produced following removal of duplicated contigs with Purge_haplotigs v.1.0.4 [135] with input generated from the long read aligner, minimap2 (https://github.com/lh3/minimap2) [63]. Estimated coverage and completeness was assessed by mapping rate of aligned filtered short Illumina PE reads to the draft *L. glycinivorella* contig assembly using bwa v.0.7.17-r1188 [136] with default settings, and coverage depth defined using the -depth command of SamTools v.1.9 (https://github.com/samtools/samtools) [137]. Representation and completeness of the contig assembly was assessed using Benchmarking Universal Single-Copy Orthologs (BUSCO) v.5.2.2 against the set of 5,286 orthologs in lepidoptera_odb10 [138–140] using default parameters with the MetaEuk search algorithm [141].

### Scaffolding

Chromatin Conformation Capture (Hi-C) libraries were prepared by Benagen Tech Solutions Ltd. (Wuhan, China) according to methods described previously [142]. For this, chromatin was cross linked to DNA by formaldehyde treatment in vitro, then DNA digested with *Dpn*II, and the 5’ overhangs blunted by DNA pol I Kelnow fragment incorporation of biotinylated nucleotides. Following ligation of proximal blunted DNA ends and removal of histone crosslinks, genomic DNA was purified to remove protein and unincorporated biotinylated nucleotides. These purified ligation products were then sheared into 350 to 700 bp fragments, and short read libraries constructed using the NEB Next Ultra Library Prep kit (New England BioLabs, Ipswich, MA, USA), followed by purification using streptavidin beads and PCR enrichment. The library was quantified using Quant-iT™ PicoGreen™ dsDNA Assay Kit (Thermo-Fisher, Wilmington, DE, USA) on a Qubit® 2.0 fluorometer (Thermo-Fisher) and a qPCR-based method [130]. Library insert sizes were estimated following separation on an Agilent 2100 BioAnalyzer (Agilent Technologies Inc.). PE 150 bp reads were then generated from library inserts on a single S2 flow cell lane of an Illumina NovaSeq 6000 sequencer (Illumina).

All post-processing of Hi-C read data was performed using HiCUP v.0.7.2 [143] to generate di-tags (PE reads located on different contigs) and used for downstream scaffolding. Specifically, HiCUP v.0.7.2 was used to trim raw reads of sequencing adapters, linker sequence, and low-quality nucleotides (*q* < 30) followed by truncation of reads to sequence upstream of the *Dpn*II site. Trimmed reads were then aligned to the draft contig assembly using Bowtie 2 [144]. Any di-tags resulting from self-circularization, representing dangling ends or internal fragments (map to the same *Dpn*II site), too distant from a *Dpn*II site in the primary contig assembly, or repetitive PCR duplicates were also removed. The valid unique aligned reads were then used to predict inter- and intra-contig interactions, and define putative order and orientation along chromosomes. Juicebox v1.11.08 (https://github.com/phasegenomics/juicebox_scripts) was used to manually curate the contact-map wherein mis-joins were eliminated, inverted contigs re-oriented, and contigs joined into scaffolds.

The final set of *L. glycinivorella* scaffolds were aligned to 28 chromosome-level scaffolds of the 630.6 Mb *Cydia splendana* (Lepidoptera: Tortricidae; Family Olethreutinae) genome assembly ilCydSple1.1 (Welcome Sager Institute, unpublished; GenBank: GCA_910591565.1 WGS Project: CAJUYE01) using Nucmer in the MUMmer4 package [145]. Nucmer alignments were generated using default parameters except anchored using maximal unique matching sequence (–mum) of 65 nt, followed by use of *delta-filter* to filter the subsequent delta (alignment) file for minimum identity (-i) of 75, minimum length (-l) 1000, minimum uniqueness (-u) of 50, and maximum alignment overlap (-o) of 100. Coordinates of the filtered delta file were output using the MUMmer4 *show-coords* application, and 2D plots generated from *mummerplot* in postscript format. Completeness of this final scaffolded assembly was assessed against the lepidoptera_odb10 gene set using BUSCO v.5.2.2 as described above. For comparison, BUSCO scores were similarly produced for other assemblies from species in the lepidopteran Family Tortricidae: *Cydia splendana*, ilCydSple1.1, *Pammene fasciana*, ilPamFasc1.1 (GenBank: GCA_911728535.1), *Notocelia uddmanniana*, ilNotUddm1.1 (GenBank: GCA_905163555.1), and *Apotomis turbidana*, ilApoTurb1.1 (GenBank: GCA_905147355.1).

### Gene annotation

The final set of chromosome-assigned *L. glycinivorella* scaffolds were submitted to the National Center for Biotechnology Information (NCBI) for automated eukaryotic genome annotation pipeline [146]. Evidence used to predict NCBI reference sequence (RefSeq) gene models were based on 135.4 million RNA-seq reads in the NCBI Sequence Read Archive (SRA) runs SRR5985984—SRR5985989 previously generated from 1st instar *L. glycinivorella* larvae [147, 148]. Additional evidence was generated in this study as life-stage specific reads (Libraries Lgly00 to Lgly06c; Table S1) from samples of eggs, larvae, and adults collected from fields of cultivated *G. max* at JAAS-NARC during July and early August, 2021 (*n* = 5; BioSamples SAMN24169476 to SAMN24169480). For the latter, samples were immediately flash frozen in liquid nitrogen in the field, then sent to Benagen Tech Solutions Ltd (Wuhan, China) for RNA extraction, construction of indexed RNA-seq libraries, and generation of 150 bp PE read data on a single S4 flow cell lane of a NovaSeq 6000 (Illumina). De novo repeat detection relied upon NCBI running of WindowMasker [149].

Further phylogenetic analyses performed for putative candidate Bt resistance genes, membrane-bound aminopeptidase N (*apn*) and alkaline phosphatase (*alp*), where orthology was uncertain based on RefSeq structural annotation. The Clustal W algorithm was used in the MEGA8.0 alignment utility [150] to construct a multiple protein sequence alignment of 16 APN proteins from *B. mori* (*n* = 16; [107]), *O. nubilalis* (*n* = 9; [109, 112]) and 27 *L. glycinivorella* RefSeq protein models with putative alanyl-aminopeptidase annotations encoded by 20 RefSeq gene models. A query of the Conserved Domain Database with the *O. nubilalis* APN1 (accession ACJ64827.1) predicted a 460 aa Peptidase M1 domain that was used to define the orthologous region in the multiple sequence alignment. Variation in the Peptidase M1 domain was subsequently used for subsequent phylogenetic analysis. The BIC score was maximized for this trimmed alignment in the LG model of protein sequence evolution [151] with an empirically-determined gamma distribution (LG + G), and MEGA8.0 [150] used to construct a phylogeny from among sites with ≥ 80% representation among aligned residues using Maximum Likelihood (ML) based on a consensus of 1,000 bootstrap pseudo-replicates.

For the *L*. *glycinivorella* gene LOC125230228 (transcript isoforms XM_048135319.1 and XM_048135320.1 encoding proteins XP_047991276.1 and XP_047991277.1, respectively), amino acid sequence homology was predicted based on simple alignment with *Heliothis virescens* mALPs (HvmALP) orthologs. Specifically, *L*. *glycinivorella* XP_047991276.1 and XP_047991277.1 were aligned with *Heliothis virescens* mALP accessions EU729322.1 (HvmALP1) and EU729323.1 (HvmALP1; [78]) using the Clustal W algorithm with default parameters and percent identities used to define orthology among putative isoforms.

### Larval transcriptome response to feeding on transgenic G. max expressing Cry1Ac

The *G. max* cultivar, GP03-8–23, that express 3.75 μg/g of the Bt Cry1Ac pesticidal protein [61] and a conventional (non-Bt) cultivar were grown to R3 stage in a greenhouse at JAAS at 24 ± 1 °C, 60% relative humidity (RH), and 16:8 h light:dark (L:D). *Leguminivora glycinivorella* adults were collected from non-Bt *G. max* fields at JAAS-NARC in July 2021, placed into a cage, and eggs collected. Subsequent neonates were fed detached conventional (non-Bt) *G. max* leaves placed on moistened filter paper in 15-cm Petri plates and incubated in a growth chamber (26 ± 1 °C, 60–70% RH, and 16:8 L:D photoperiod). Second instars were transferred to Cry1Ac GP03-8–23 or non-Bt *G. max* plants (treatments), one larva per plant, across three replicates of 20 plants per treatment (60 plants per treatment; 120 total). Larvae were allowed to feed for 48 h. Recovered larvae were pooled from within each of the 3 replicates for Cry1Ac GP03-8–23 and non-Bt treatments (*n* = 7 larvae per treatment: *n* = 42 total larvae) for a total of 6 pools (Table S1; SAMN24169481 to SAMN24169486). Pools were flash frozen in liquid nitrogen and shipped to Benagen Tech Solutions Ltd. (Wuhan, China) where RNA was extracted and indexed RNA-seq libraries constructed for each Cry1Ac GP03-8–23 (Glyc05a_1Ac to Glyc05c_1Ac) and non-Bt treatment (Lgly06a_nBt to Lgly06a_nBt). Equimolar amounts of each library loaded on a single S4 lane of a NovaSeq 6000 (Illumina) on which 150 bp PE reads were generated.

Raw non-normalized fastq formatted read data were trimmed to remove nucleotide sequence with a *q* < 20 and Illumina adapters using Trimmomatic v0.39 [152]. Only PE reads from each library that survived filtering were used for generating pseudoalignments to *L. glycinivorella* transcript models (NCBI accession GCF_023078275.1) with Kallisto v0.46.1 [153] (parameters: –fragment-length = 200; –sd 20; –bootstrap-samples = 100, –seed = 42). Estimated read counts for each transcript pseudoalignment was used to predict differential gene expression (DGE; aggregated estimate of transcript isoforms), differential alternative splicing (DAS), and differential transcript use (DTU) events between treatments of *L. glycinivorella* fed Cry1Ac cultivar GP03-8–23 (Lgly05a_1Ac to Lgly 05c_1Ac) vs. non-Bt cultivar (Lgly06a_nBt to Lgly06a_nBt; Table S1) using the 3D RNA-seq package v 1.0.0 (https://github.com/wyguo/ThreeDRNAseq) [154] run in R 4.4.2 [155] via the integrated development environment, Rstudio 2022.07.2 + 576 [156]. A transcript mapping file consisting of ≥ 1 RefSeq mRNA model (XP_) from each gene (LOC|GeneID) was provided as input [23,735 transcripts (XM_) and 2,055 non-coding RNAs (XR_) from 17,151 RefSeq gene models]. Pre-processing involved conversion of read counts to length-scaled transcripts per million (lengthScaledTPM) [156], and filtered to remove genes and transcripts with a decreasing mean–variance trend; those in which ≥ 1 of 6 replicates with counts per million (CPM) ≥ 1. Read counts were normalized to log_2_CPM via trimmed mean of M-values (TMM) [157]. Then counts were corrected for batch outlier effects using the RUVr package [158] run in R 4.4.2 [155], and Principal Component Analysis (PCA) used to assess corrected mean log_2_CPM estimates among genes (aggregate of estimates for all transcripts for each gene) from each replicate along principal coordinates 1 (PC1) and 2 (PC2). Limma-voom [159] was then used to estimate the log_2_(fold-change) of transcript abundances based on CPM values between treatment groups [Cry1Ac (Lgly05a_1Ac to Lgly 05c_1Ac) vs. non-Bt (Lgly06a_nBt to Lgly06a_nBt)] and determined a false discovery rate (FDR) adjusted for multiple comparisons using the Benjamini-Hochberg (BH) method [108]. Significance for DGE was defined at an FDR threshold ≤ 0.05 and log_2_(fold-change) ≥ 2.0. DAS and DTU events were predicted based on change in percentage spliced transcripts (∆PS; change in ratio of mean transcript abundances weighted by the mean abundance for all transcripts for the given gene) [160] as calculated in the 3D RNA-seq package [154]. In brief, ∆PS was calculated for genes with > 1 transcript and DAS only events defined as instances where proportional changes in transcript abundances are predicted without corresponding significant changes in expression when all transcript isoform levels are considered. DTU (DGE + DAS) events are those when significant changes in overall gene expression and relative transcript abundances are predicted. Significance of DAS and DTU estimates were determined using an *F*-test with a BH adjusted FDR ≤ 0.05 and ∆PS ≥ 0.1.

Functional annotation was performed for significant differentially expressed RefSeq transcripts by prediction of protein domains by searching the Pfam-A database [161] with PfamScan (https://www.ebi.ac.uk/Tools/pfa/pfamscan/) using each corresponding RefSeq proteins as the query, with results filtered for *E*-values ≤ 10^–10^. To avoid biased representation in instances when multiple transcripts were derived from same gene (LOC/GeneID), the longest RefSeq protein model was used to query Pfam-A. Pfam domains were then used to retrieve curated gene ontology (GO) terms and perform enrichment analyses at GO level 2 using the dcGOR 1.0.6 package [162] based *Z*-scores calculated on hypergeometric distributions of terms and a BH significance threshold (FDR) ≤ 1.0^–4^ applied for cellular component (CC) and molecular function (MF), and ≤ 1.0^–6^ biological process (BP) category terms. GOplot [163] was used to construct a bubble plot (GObubble) where -log(FDR) from GO enrichment was plotted against a *Z*-score that took into account expression bias among DEGs [Z-score = (count up-regulated – down-regulated genes)/sqrt(total number of genes)] with positive and negative values indicative of greater proportional of up- and down-regulated genes in a given GO category, respectively. Size of bubbles proportional to number of DEGs in each category.

### Supplementary Information

**Additional file 1. Supplementary Figure S1a-c.**

**Additional file 2: Supplementary Figure S2.** NucmerSummaryStats

**Additional file 3: Supplementary Figure S3.** RNAseqPreProc_MeanVarPlots

**Additional file 4: Supplementary Figure S4.** RNAseq_Normalization

**Additional file 5: Supplementary Figure S5.** APNphylogeny

**Additional file 6: Supplementary Table S1.**

**Additional file 7: Supplementary Table S2.** ScaffoldAssignments

**Additional file 8: Supplementary Table S3.** Updated_Nucmer_show-coords_LglyVsCydSple

**Additional file 9: Supplementary Table S4.** NucmerSummaryV2

**Additional file 10: Supplementary Table 5a-e.** DE gene testing statistics+13902+Pfam+GO_05222023

**Additional file 11: Supplementary Table S6.** DE transcripts testing statistics

**Additional file 12: Supplementary Table S7.** GOsAllLevels_Significant.

## Data Availability

All read data generated in this study are under PRJNA759210 (co-listed under the i5K Umbrella Project, PRJNA163973). This includes sequence read archive (SRA) submissions for whole genome sequence reads (SRR15680761 to SRR15680763), and RNA-seq reads generated across developmental stages used for evidence-based gene predictions (SRR17269421 to SRR17269426) and prediction of differential expression (SRR17269416 to SRR17269424). Scaffolds from the final assembly LegGlyc1.1 are available in DDBJ/ENA/GenBank in accession JAKXMO000000000 and RefSeq genome assembly accession GCF_023078275.1 (WGS Project: JAKXMO01). Information from Annotation Release 100 (https://www.ncbi.nlm.nih.gov/genome/annotation_euk/Leguminivora_glycinivorella/100/) for transcript, CDS and protein sequences can be downloaded via FTP NCBI. The primary assembly and annotations are also available for view through WebApollo at the i5k Workspace@NAL  [164].
