# Supplementary material for: A chromosome-level genome assembly of the soybean pod borer: insights into larval transcriptional response to transgenic soybean expressing the pesticidal Cry1Ac protein
Source: BMC Genomics. 2024 Apr 9;25:355. doi: 10.1186/s12864-024-10216-2 (PMC11005160; doi:10.1186/s12864-024-10216-2)
Supplement: Supplementary file 1 — Additional file 1. Supplementary Figure S1a-c. [file 12864_2024_10216_MOESM1_ESM.docx]

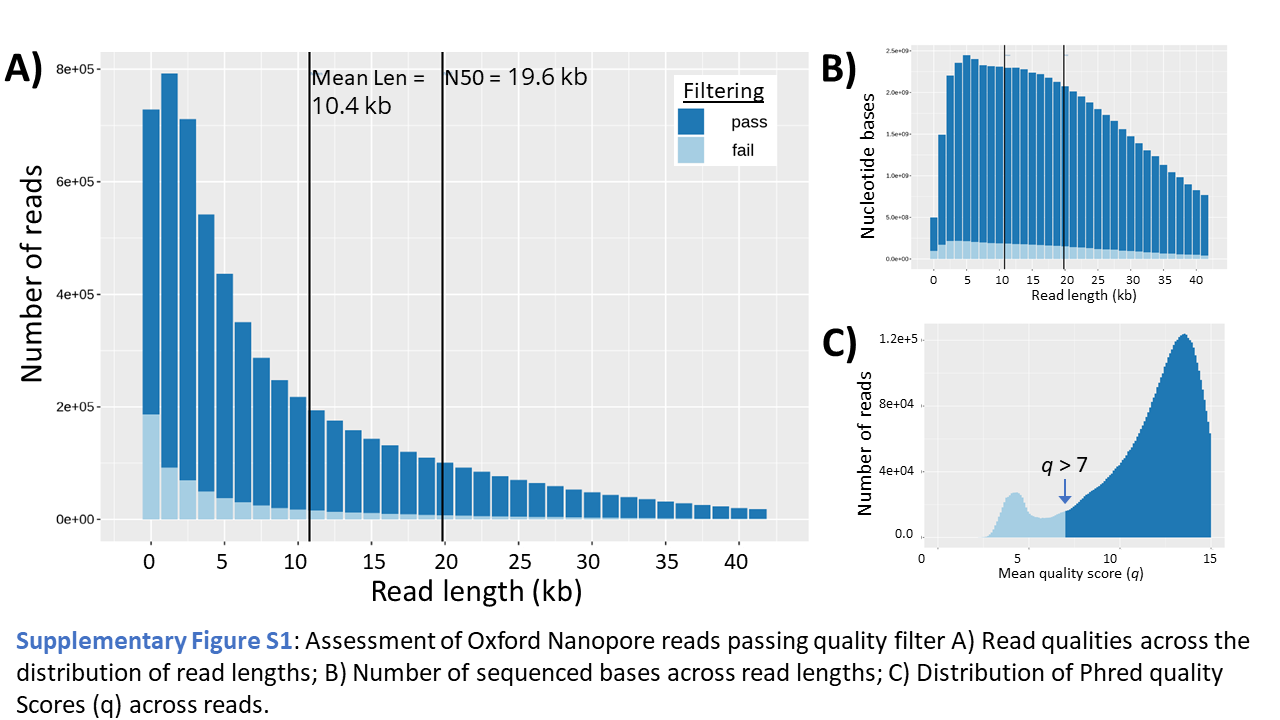


**Supplementary Fig. S1**: Assessment of Oxford Nanopore reads passing quality filter A) Read qualities across the distribution of read lengths; B) Number of sequenced bases across read lengths; C) Distribution of Phred quality Scores (*q*) across reads.
