# Supplementary material for: A chromosome-level genome assembly of the soybean pod borer: insights into larval transcriptional response to transgenic soybean expressing the pesticidal Cry1Ac protein
Source: BMC Genomics. 2024 Apr 9;25:355. doi: 10.1186/s12864-024-10216-2 (PMC11005160; doi:10.1186/s12864-024-10216-2)
Supplement: Supplementary file 2 — Additional file 2: Supplementary Figure S2. NucmerSummaryStats [file 12864_2024_10216_MOESM2_ESM.docx]

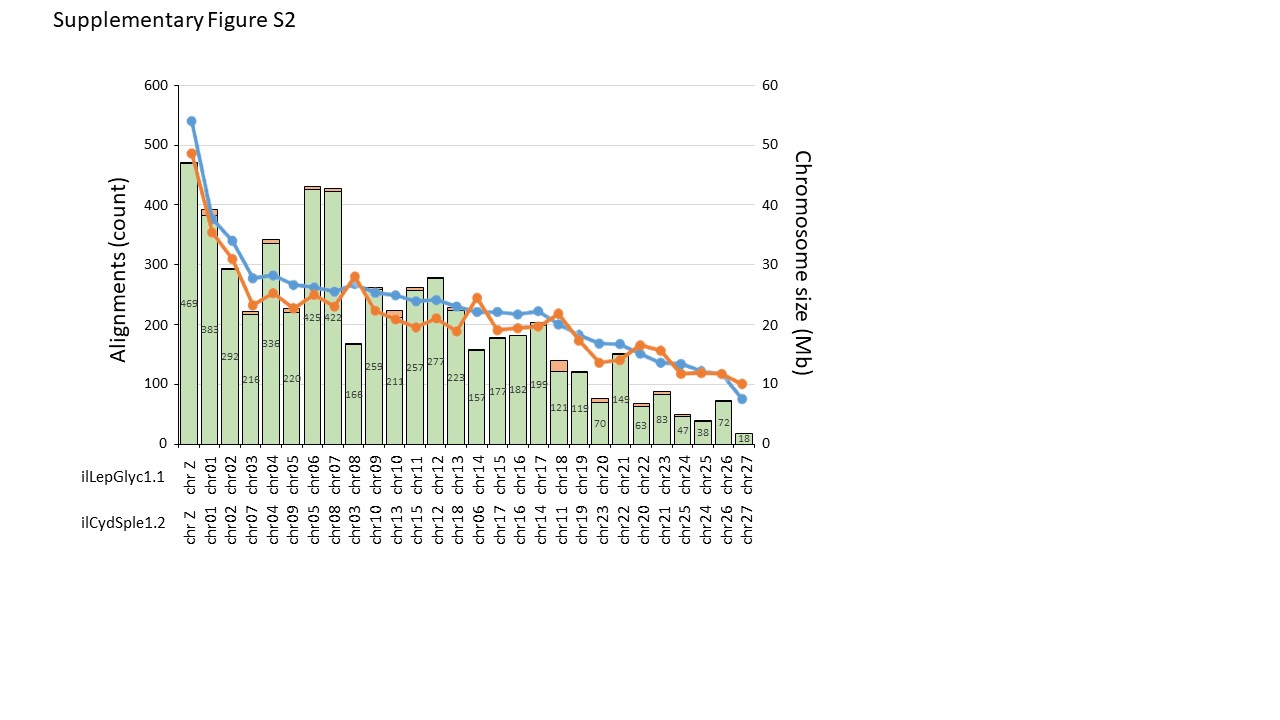


**Supplementary Fig. S2**. Orthology between chromosomes assigned within National Center for Biotechnology Information (NCBI) RefSeq assemblies for Leguminivora glycinivorella, ilLegGlyc1.1, and *Cydia Splenada*, ilCydSple1.2. Bar graph indicates count of alignments resulting from query of ilLegGlyc1.1 to ilCydSple1.2 (green = putative orthologous alignments between chromosome; pink = putative misalignments). Inset line graph shows corresponding chromosome lengths for ilLegGlyc1.1 (blue) and ilCydSple1.2 (orange).
