# Supplementary material for: A chromosome-level genome assembly of the soybean pod borer: insights into larval transcriptional response to transgenic soybean expressing the pesticidal Cry1Ac protein
Source: BMC Genomics. 2024 Apr 9;25:355. doi: 10.1186/s12864-024-10216-2 (PMC11005160; doi:10.1186/s12864-024-10216-2)
Supplement: Supplementary file 3 — Additional file 3: Supplementary Figure S3. RNAseqPreProc_MeanVarPlots [file 12864_2024_10216_MOESM3_ESM.docx]

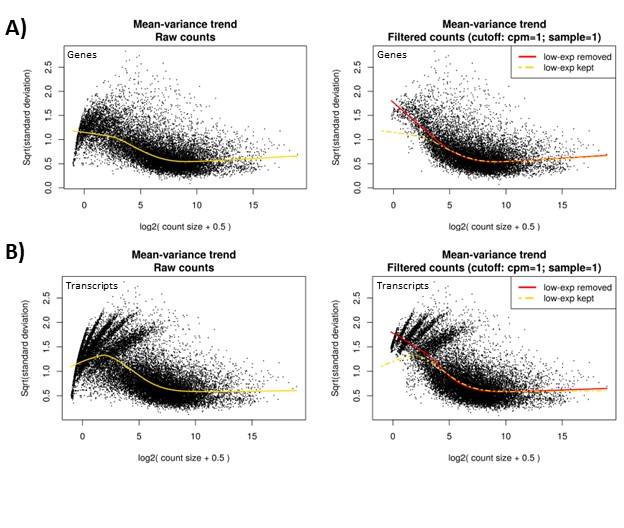


**Fig. S3**: Dispersion of raw and filtered read counts about empirical mean for comparisons of triplicate RNA-seq data aligned to **A)** genes and **B)** transcripts. Filter imposed to remove low-expressing transcripts from read alignments; ≥1 of 6 replicates and with estimated counts per million (CPM) ≥
