# Supplementary material for: A chromosome-level genome assembly of the soybean pod borer: insights into larval transcriptional response to transgenic soybean expressing the pesticidal Cry1Ac protein
Source: BMC Genomics. 2024 Apr 9;25:355. doi: 10.1186/s12864-024-10216-2 (PMC11005160; doi:10.1186/s12864-024-10216-2)
Supplement: Supplementary file 4 — Additional file 4: Supplementary Figure S4. RNAseq_Normalization [file 12864_2024_10216_MOESM4_ESM.docx]

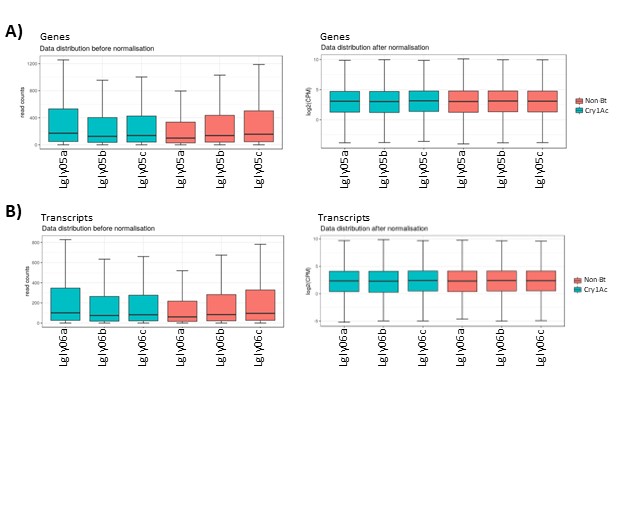


**Supplementary Fig. S4** Distribution of filtered read counts for **A)** gene and **B)** transcript levels (filtered for counts per million > 1 and sample number > 1 among 6 replicated RNA-seq datasets; **Fig. S3**), before and after normalization by log_2_(counts per million), log_2_(CPM).
