# Supplementary material for: A chromosome-level genome assembly of the soybean pod borer: insights into larval transcriptional response to transgenic soybean expressing the pesticidal Cry1Ac protein
Source: BMC Genomics. 2024 Apr 9;25:355. doi: 10.1186/s12864-024-10216-2 (PMC11005160; doi:10.1186/s12864-024-10216-2)
Supplement: Supplementary file 5 — Additional file 5: Supplementary Figure S5. APNphylogeny [file 12864_2024_10216_MOESM5_ESM.docx]

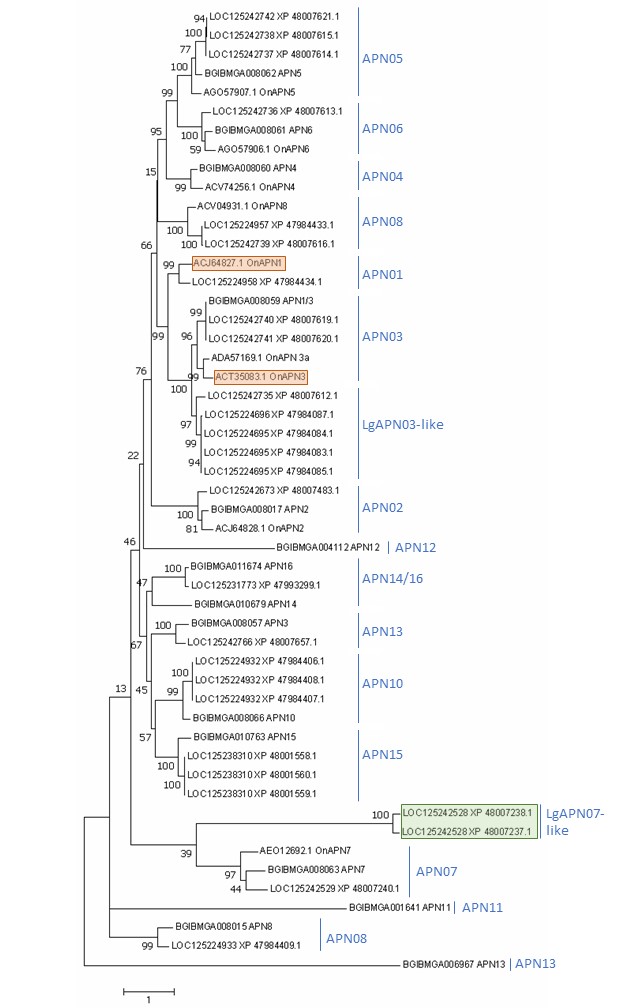


**Supplementary Fig. S5** Phylogenetic reconstruction of the *Leguminivora glycinivorella* aminopeptidase N gene family incorporating RefSeq transcripts (“XM_” prefix) derived from each gene model (“LOC” prefix). Relationships shown with respect to orthologs from *Bombyx mori* (BGIBMGA) and *Ostrinia nubilalis* (On) in a consensus tree that maximized the log-likelihood at -27211.74 with a gamma shape parameter = 4.0164 constructed from a 434-residue consensus amino acid sequence alignment. Proteins XP_48003237.1 and XP_48003238.1 encoded by the *L. glycinivorella* aminopeptidase N-like gene LOC125242528 (Green box) that is predicted to be significantly up-regulated in Cry1Ac-exposed larvae clusters with a putative APN7 clad. Clades for insecticidal *Bacillus thuringiensis* protein receptors, APN1 and 3 (orange boxes), previously shown to mediate Cry1 resistance when down-regulated (Coates et al. 2013).
