# Supplementary material for: A chromosome-level genome assembly of the soybean pod borer: insights into larval transcriptional response to transgenic soybean expressing the pesticidal Cry1Ac protein
Source: BMC Genomics. 2024 Apr 9;25:355. doi: 10.1186/s12864-024-10216-2 (PMC11005160; doi:10.1186/s12864-024-10216-2)
Supplement: Supplementary file 6 — Additional file 6: Supplementary Table S1. [file 12864_2024_10216_MOESM6_ESM.docx]

**Table S1** Sequence read data used for assembly, scaffolding, and annotation of the Leguminivora glycinivorella genome, ilLegGlyc1.1 (BioProject PRJNA759210; Locus Tag Prefix: K7X69).

|  |  |  |  |  |  |  | Raw reads |  | Trimmed reads | |  |  |
| --- | --- | --- | --- | --- | --- | --- | --- | --- | --- | --- | --- | --- |
| Assembly | Library ID | Source |  | BioSample |  | Type | Count (M) |  | Count (M) | Gb |  | SRA accession |
|  | LglySR | Larva#1 |  | SAMN21160035 |  | PE | 386.7 |  | 386.7 | 58.0 |  | SRR15680763 |
|  | LglyHiC | Larva#1 |  | SAMN21160035 |  | PE | 307.5 |  | 302.0 | 45.1 |  | SRR15680762 |
|  | LglyNP | Larva#2 |  | SAMN21160035 |  | SE | 6.4 |  | 5.7 | 61.6 |  | SRR15680761 |
|  |  |  |  |  |  |  |  |  |  |  |  |  |
| Annotation | Lgly00_lar | 4^th^ instar (*n* = 1) |  | SAMN24169476 |  | PE | 36.1 |  | 34.7 | 10.2 |  | SRR17269426 |
|  | Lgly01_af | Adult female (*n* = 1) |  | SAMN24169477 |  | PE | 23.3 |  | 23.1 | 6.9 |  | SRR17269425 |
|  | Lgly02_am | Adult male (*n* = 1) |  | SAMN24169478 |  | PE | 24.7 |  | 24.4 | 7.3 |  | SRR17269423 |
|  | Lgly03_egg | Eggs (*n* = 40) |  | SAMN24169479 |  | PE | 22.7 |  | 22.4 | 6.7 |  | SRR17269422 |
|  | Lgly04_mg | 5^th^ instar gut (*n* = 30) |  | SAMN24169480 |  | PE | 23.6 |  | 23.4 | 7.0 |  | SRR17269421 |
|  |  |  |  |  |  |  |  |  |  |  |  |  |
| Bt exposure | Lgly05a_1Ac-1 | 3^rd^ instars; 2-day exposure to Cry1Ac *Glycine max* (*n* = 7) |  | SAMN24169481 |  | PE | 24.9 |  | 24.7 | 7.3 |  | SRR17269420 |
|  | Lgly05b_1Ac-2 |  |  | SAMN24169485 |  | PE | 21.5 |  | 21.3 | 6.3 |  | SRR17269419 |
|  | Lgly05c_1Ac-3 |  |  | SAMN24169483 |  | PE | 20.3 |  | 20.1 | 5.9 |  | SRR17269418 |
|  | Lgly06a_nBt-1 | 3^rd^ instars; 2-day exposure to non-Bt *Glycine max* (*n* = 7) |  | SAMN24169484 |  | PE | 20.0 |  | 19.8 | 5.9 |  | SRR17269417 |
|  | Lgly06b_nBt-2 |  |  | SAMN24169485 |  | PE | 23.9 |  | 23.7 | 7.0 |  | SRR17269416 |
|  | Lgly06c_nBt-3 |  |  | SAMN24169486 |  | PE | 23.4 |  | 23.1 | 6.9 |  | SRR17269424 |
