# Supplementary material for: A chromosome-level genome assembly of the soybean pod borer: insights into larval transcriptional response to transgenic soybean expressing the pesticidal Cry1Ac protein
Source: BMC Genomics. 2024 Apr 9;25:355. doi: 10.1186/s12864-024-10216-2 (PMC11005160; doi:10.1186/s12864-024-10216-2)
Supplement: Supplementary file 7 — Additional file 7: Supplementary Table S2. ScaffoldAssignments [file 12864_2024_10216_MOESM7_ESM.docx]

**Table S2:** Size and component contigs within Leguminivora glycinivorella scaffolded chromosomes (*n* = 28; 27 autosomes + Z chromosome).

|  | Accessions | |  |  |
| --- | --- | --- | --- | --- |
| Chromosome | Sequence | RefSeq | Len (bp) | Contigs |
| Chr01 | [CM041121.1](https://www.ncbi.nlm.nih.gov/nuccore/CM041121.1) | [NC_062971.1](https://www.ncbi.nlm.nih.gov/nuccore/NC_062971.1) | 38,502,580 | 11 |
| Chr02 | [CM041122.1](https://www.ncbi.nlm.nih.gov/nuccore/CM041122.1) | [NC_062972.1](https://www.ncbi.nlm.nih.gov/nuccore/NC_062972.1) | 34,640,967 | 7 |
| Chr03 | [CM041123.1](https://www.ncbi.nlm.nih.gov/nuccore/CM041123.1) | [NC_062973.1](https://www.ncbi.nlm.nih.gov/nuccore/NC_062973.1) | 28,204,004 | 11 |
| Chr04 | [CM041124.1](https://www.ncbi.nlm.nih.gov/nuccore/CM041124.1) | [NC_062974.1](https://www.ncbi.nlm.nih.gov/nuccore/NC_062974.1) | 28,698,277 | 8 |
| Chr05 | [CM041125.1](https://www.ncbi.nlm.nih.gov/nuccore/CM041125.1) | [NC_062975.1](https://www.ncbi.nlm.nih.gov/nuccore/NC_062975.1) | 27,042,002 | 9 |
| Chr06 | [CM041126.1](https://www.ncbi.nlm.nih.gov/nuccore/CM041126.1) | [NC_062976.1](https://www.ncbi.nlm.nih.gov/nuccore/NC_062976.1) | 26,623,903 | 12 |
| Chr07 | [CM041127.1](https://www.ncbi.nlm.nih.gov/nuccore/CM041127.1) | [NC_062977.1](https://www.ncbi.nlm.nih.gov/nuccore/NC_062977.1) | 25,900,846 | 6 |
| Chr08 | [CM041128.1](https://www.ncbi.nlm.nih.gov/nuccore/CM041128.1) | [NC_062978.1](https://www.ncbi.nlm.nih.gov/nuccore/NC_062978.1) | 27,165,122 | 12 |
| Chr09 | [CM041129.1](https://www.ncbi.nlm.nih.gov/nuccore/CM041129.1) | [NC_062979.1](https://www.ncbi.nlm.nih.gov/nuccore/NC_062979.1) | 25,672,803 | 10 |
| Chr10 | [CM041130.1](https://www.ncbi.nlm.nih.gov/nuccore/CM041130.1) | [NC_062980.1](https://www.ncbi.nlm.nih.gov/nuccore/NC_062980.1) | 25,267,037 | 10 |
| Chr11 | [CM041131.1](https://www.ncbi.nlm.nih.gov/nuccore/CM041131.1) | [NC_062981.1](https://www.ncbi.nlm.nih.gov/nuccore/NC_062981.1) | 24,202,123 | 8 |
| Chr12 | [CM041132.1](https://www.ncbi.nlm.nih.gov/nuccore/CM041132.1) | [NC_062982.1](https://www.ncbi.nlm.nih.gov/nuccore/NC_062982.1) | 24,472,463 | 11 |
| Chr13 | [CM041133.1](https://www.ncbi.nlm.nih.gov/nuccore/CM041133.1) | [NC_062983.1](https://www.ncbi.nlm.nih.gov/nuccore/NC_062983.1) | 23,289,756 | 8 |
| Chr14 | [CM041134.1](https://www.ncbi.nlm.nih.gov/nuccore/CM041134.1) | [NC_062984.1](https://www.ncbi.nlm.nih.gov/nuccore/NC_062984.1) | 22,349,846 | 7 |
| Chr15 | [CM041135.1](https://www.ncbi.nlm.nih.gov/nuccore/CM041135.1) | [NC_062985.1](https://www.ncbi.nlm.nih.gov/nuccore/NC_062985.1) | 22,349,913 | 8 |
| Chr16 | [CM041136.1](https://www.ncbi.nlm.nih.gov/nuccore/CM041136.1) | [NC_062986.1](https://www.ncbi.nlm.nih.gov/nuccore/NC_062986.1) | 21,949,049 | 9 |
| Chr17 | [CM041137.1](https://www.ncbi.nlm.nih.gov/nuccore/CM041137.1) | [NC_062987.1](https://www.ncbi.nlm.nih.gov/nuccore/NC_062987.1) | 22,478,868 | 9 |
| Chr18 | [CM041138.1](https://www.ncbi.nlm.nih.gov/nuccore/CM041138.1) | [NC_062988.1](https://www.ncbi.nlm.nih.gov/nuccore/NC_062988.1) | 20,231,578 | 8 |
| Chr19 | [CM041139.1](https://www.ncbi.nlm.nih.gov/nuccore/CM041139.1) | [NC_062989.1](https://www.ncbi.nlm.nih.gov/nuccore/NC_062989.1) | 18,379,626 | 11 |
| Chr20 | [CM041140.1](https://www.ncbi.nlm.nih.gov/nuccore/CM041140.1) | [NC_062990.1](https://www.ncbi.nlm.nih.gov/nuccore/NC_062990.1) | 16,913,803 | 6 |
| Chr21 | [CM041141.1](https://www.ncbi.nlm.nih.gov/nuccore/CM041141.1) | [NC_062991.1](https://www.ncbi.nlm.nih.gov/nuccore/NC_062991.1) | 16,800,200 | 5 |
| Chr22 | [CM041142.1](https://www.ncbi.nlm.nih.gov/nuccore/CM041142.1) | [NC_062992.1](https://www.ncbi.nlm.nih.gov/nuccore/NC_062992.1) | 15,140,355 | 6 |
| Chr23 | [CM041143.1](https://www.ncbi.nlm.nih.gov/nuccore/CM041143.1) | [NC_062993.1](https://www.ncbi.nlm.nih.gov/nuccore/NC_062993.1) | 13,540,656 | 10 |
| Chr24 | [CM041144.1](https://www.ncbi.nlm.nih.gov/nuccore/CM041144.1) | [NC_062994.1](https://www.ncbi.nlm.nih.gov/nuccore/NC_062994.1) | 13,366,367 | 3 |
| Chr25 | [CM041145.1](https://www.ncbi.nlm.nih.gov/nuccore/CM041145.1) | [NC_062995.1](https://www.ncbi.nlm.nih.gov/nuccore/NC_062995.1) | 12,132,900 | 4 |
| Chr26 | [CM041146.1](https://www.ncbi.nlm.nih.gov/nuccore/CM041146.1) | [NC_062996.1](https://www.ncbi.nlm.nih.gov/nuccore/NC_062996.1) | 11,646,544 | 3 |
| Chr27 | [CM041147.1](https://www.ncbi.nlm.nih.gov/nuccore/CM041147.1) | [NC_062997.1](https://www.ncbi.nlm.nih.gov/nuccore/NC_062997.1) | 7,314,981 | 3 |
| ChrZ | [CM041148.1](https://www.ncbi.nlm.nih.gov/nuccore/CM041148.1) | [NC_062998.1](https://www.ncbi.nlm.nih.gov/nuccore/NC_062998.1) | 55,408,930 | 10 |
| Unplaced |  | Unplaced | 7,738,113 | 12 |
|  |  |  | 657,423,512 | 237 |
| GenBank scaffold accession: JAKXMO000000000  GenBank assembly accession: GCA_023078275.1 | | | | |
