# Supplementary material for: A chromosome-level genome assembly of the soybean pod borer: insights into larval transcriptional response to transgenic soybean expressing the pesticidal Cry1Ac protein
Source: BMC Genomics. 2024 Apr 9;25:355. doi: 10.1186/s12864-024-10216-2 (PMC11005160; doi:10.1186/s12864-024-10216-2)
Supplement: Supplementary file 9 — Additional file 9: Supplementary Table S4. NucmerSummaryV2 [file 12864_2024_10216_MOESM9_ESM.docx]

**Supplementary Table S3**. Alignment of chromosomes (chr) assigned within assemblies for tortricid moths. Results of querying the assembly for Leguminivora glycinivorella, ilLegGlyc1.1, sequences against that of the reference, *Cydia Splenada*, ilCydSple1.2. National Center for Biotechnology Information (NCBI) accession and length (Mb) provided for putative orthologous chromosomes between ilLegGlyc1.1 and ilCydSple1.2: Evidence shown for alignments’ total number (Count) and subset that are putatively orthologous (Ortho; majority for respective chromosome pairs), percent identity (% ID), and length (bp).

|  |  |  |  |  |  |  |  | Alignment of ilLepGlyc1.1 with respect to reference ilCydSple1.2 | | | | | | | | | |
| --- | --- | --- | --- | --- | --- | --- | --- | --- | --- | --- | --- | --- | --- | --- | --- | --- | --- |
| ilLegGlyc1.1 | | |  | ilCydSple1.2 | | |  | Total |  | Ortho |  | Ortho % ID | |  | Ortho lengths | | |
| Chr | Accession | Len (Mb) |  | Chr | Accession | Len (Mb) |  | Counts |  | Counts |  | Mean | SD |  | Mean | SD | Total (bp) |
| chr Z | NC_062998.1 | 55.41 |  | chr Z | OU342871.1 | 49.82 |  | 469 |  | 467 |  | 89.52 | 2.96 |  | 1710.87 | 939.33 | 798,974 |
| chr01 | NC_062971.1 | 38.50 |  | chr01 | OU342872.1 | 36.11 |  | 383 |  | 374 |  | 90.05 | 2.61 |  | 1876.32 | 1149.93 | 701,743 |
| chr02 | NC_062972.1 | 34.64 |  | chr02 | OU342873.1 | 31.49 |  | 292 |  | 291 |  | 89.76 | 3.01 |  | 1647.82 | 736.02 | 481,162 |
| chr03 | NC_062973.1 | 28.20 |  | chr07 | OU342878.1 | 23.52 |  | 216 |  | 210 |  | 89.67 | 2.82 |  | 1935.07 | 1167.46 | 406,364 |
| chr04 | NC_062974.1 | 28.70 |  | chr04 | OU342875.1 | 25.63 |  | 336 |  | 330 |  | 89.58 | 2.83 |  | 1629.38 | 647.04 | 537,694 |
| chr05 | NC_062975.1 | 27.04 |  | chr09 | OU342880.1 | 22.98 |  | 220 |  | 213 |  | 89.57 | 2.79 |  | 1731.21 | 829.68 | 368,747 |
| chr06 | NC_062976.1 | 26.62 |  | chr05 | OU342876.1 | 25.31 |  | 425 |  | 420 |  | 89.84 | 2.77 |  | 1730.21 | 863.94 | 726,689 |
| chr07 | NC_062977.1 | 25.90 |  | chr08 | OU342879.1 | 23.32 |  | 422 |  | 416 |  | 89.78 | 2.90 |  | 1767.52 | 969.10 | 737,055 |
| chr08 | NC_062978.1 | 27.17 |  | chr03 | OU342874.1 | 28.51 |  | 166 |  | 164 |  | 89.26 | 2.81 |  | 1830.38 | 984.47 | 300,182 |
| chr09 | NC_062979.1 | 25.67 |  | chr10 | OU342881.1 | 22.56 |  | 259 |  | 257 |  | 89.85 | 2.85 |  | 1746.32 | 1070.78 | 448,803 |
| chr10 | NC_062980.1 | 25.27 |  | chr13 | OU342884.1 | 21.09 |  | 211 |  | 199 |  | 89.56 | 3.00 |  | 1845.80 | 1237.94 | 367,315 |
| chr11 | NC_062981.1 | 24.20 |  | chr15 | OU342886.1 | 19.69 |  | 257 |  | 252 |  | 90.33 | 1.37 |  | 1644.32 | 431.23 | 414,367 |
| chr12 | NC_062982.1 | 24.47 |  | chr12 | OU342883.1 | 21.27 |  | 277 |  | 276 |  | 89.91 | 2.95 |  | 1877.41 | 1013.15 | 518,166 |
| chr13 | NC_062983.1 | 23.29 |  | chr18 | OU342889.1 | 18.99 |  | 223 |  | 217 |  | 89.98 | 2.95 |  | 1691.27 | 778.17 | 367,006 |
| chr14 | NC_062984.1 | 22.35 |  | chr06 | OU342877.1 | 24.84 |  | 157 |  | 156 |  | 89.47 | 2.58 |  | 1738.40 | 1008.32 | 271,191 |
| chr15 | NC_062985.1 | 22.35 |  | chr17 | OU342888.1 | 19.20 |  | 177 |  | 176 |  | 89.24 | 2.66 |  | 1790.21 | 986.09 | 315,076 |
| chr16 | NC_062986.1 | 21.95 |  | chr16 | OU342887.1 | 19.55 |  | 182 |  | 182 |  | 89.38 | 2.83 |  | 1978.41 | 1218.12 | 360,070 |
| chr17 | NC_062987.1 | 22.48 |  | chr14 | OU342885.1 | 19.83 |  | 199 |  | 194 |  | 89.63 | 2.85 |  | 1711.54 | 771.37 | 332,038 |
| chr18 | NC_062988.1 | 20.23 |  | chr11 | OU342882.1 | 22.06 |  | 121 |  | 103 |  | 89.07 | 2.66 |  | 1794.13 | 952.22 | 186,589 |
| chr19 | NC_062989.1 | 18.38 |  | chr19 | OU342890.1 | 17.39 |  | 119 |  | 117 |  | 89.25 | 2.76 |  | 1818.50 | 796.10 | 212,764 |
| chr20 | NC_062990.1 | 16.91 |  | chr23 | OU342894.1 | 13.60 |  | 70 |  | 64 |  | 88.53 | 3.09 |  | 1836.41 | 733.29 | 117,530 |
| chr21 | NC_062991.1 | 16.80 |  | chr22 | OU342893.1 | 14.00 |  | 149 |  | 146 |  | 89.32 | 2.81 |  | 1818.80 | 1112.52 | 265,544 |
| chr22 | NC_062992.1 | 15.14 |  | chr20 | OU342891.1 | 16.60 |  | 63 |  | 58 |  | 88.77 | 3.27 |  | 1961.62 | 1638.51 | 113,774 |
| chr23 | NC_062993.1 | 13.54 |  | chr21 | OU342892.1 | 15.66 |  | 83 |  | 78 |  | 89.22 | 2.64 |  | 1519.54 | 613.66 | 118,524 |
| chr24 | NC_062994.1 | 13.37 |  | chr25 | OU342896.1 | 11.65 |  | 47 |  | 45 |  | 89.01 | 2.75 |  | 1600.33 | 549.92 | 72,015 |
| chr25 | NC_062995.1 | 12.13 |  | chr24 | OU342895.1 | 11.77 |  | 38 |  | 37 |  | 87.90 | 2.52 |  | 1756.29 | 797.22 | 59,714 |
| chr26 | NC_062996.1 | 11.65 |  | chr26 | OU342897.1 | 11.63 |  | 72 |  | 71 |  | 88.69 | 2.24 |  | 1768.13 | 857.06 | 125,537 |
| chr27 | NC_062997.1 | 7.31 |  | chr27 | OU342898.1 | 9.89 |  | 18 |  | 18 |  | 89.38 | 3.81 |  | 1649.22 | 691.22 | 29,686 |
|  |  | 649.69 |  |  |  | 597.96 |  | 5,651 |  | 5,531 |  |  |  |  |  |  | 9,754,319 |
